# Albinism-Causing Mutations in Recombinant Human Tyrosinase Alter Intrinsic Enzymatic Activity

**DOI:** 10.1371/journal.pone.0084494

**Published:** 2014-01-02

**Authors:** Monika B. Dolinska, Elena Kovaleva, Peter Backlund, Paul T. Wingfield, Brian P. Brooks, Yuri V. Sergeev

**Affiliations:** 1 National Eye Institute, NIH, Bethesda, Maryland, United States of America; 2 Chesapeake PERL, Savage, Maryland, United States of America; 3 Eunice Kennedy Shriver National Institute Child Health and Human Development, NIH, Bethesda, Maryland, United States of America; 4 National Institute of Arthritis and Musculoskeletal and Skin Diseases, NIH, Bethesda, Maryland, United States of America; Penn State College of Medicine, United States of America

## Abstract

**Background:**

Tyrosinase (TYR) catalyzes the rate-limiting, first step in melanin production and its gene (*TYR)* is mutated in many cases of oculocutaneous albinism (OCA1), an autosomal recessive cause of childhood blindness. Patients with reduced TYR activity are classified as OCA1B; some OCA1B mutations are temperature-sensitive. Therapeutic research for OCA1 has been hampered, in part, by the absence of purified, active, recombinant wild-type and mutant human enzymes.

**Methodology/Principal Findings:**

The intra-melanosomal domain of human tyrosinase (residues 19–469) and two OCA1B related temperature-sensitive mutants, R422Q and R422W were expressed in insect cells and produced in *T. ni* larvae. The short trans-membrane fragment was deleted to avoid potential protein insolubility, while preserving all other functional features of the enzymes. Purified tyrosinase was obtained with a yield of >1 mg per 10 g of larval biomass. The protein was a monomeric glycoenzyme with maximum enzyme activity at 37°C and neutral pH. The two purified mutants when compared to the wild-type protein were less active and temperature sensitive. These differences are associated with conformational perturbations in secondary structure.

**Conclusions/Significance:**

The intramelanosomal domains of recombinant wild-type and mutant human tyrosinases are soluble monomeric glycoproteins with activities which mirror their *in vivo* function. This advance allows for the structure – function analyses of different mutant TYR proteins and correlation with their corresponding human phenotypes; it also provides an important tool to discover drugs that may improve tyrosinase activity and treat OCA1.

## Introduction

Tyrosinase is a type 1 trans-membrane and copper-containing glycoenzyme (MIM*606933) that catalyzes the initial and rate-limiting steps of melanin pigment production in organelles called melanosomes [1], [2]. Mutations in the tyrosinase gene cause oculocutaneous albinism Type 1 (OCA1), an autosomal recessive disorder characterized by reduced melanin pigment in the hair, skin and eyes. OCA1 is further subdivided into two categories: 1) OCA1A, (MIM#203100), in which tyrosinase activity and melanin synthesis are undetectable and 2) OCA1B, (#606952) in which tyrosinase activity and melanin deposition are present, but reduced compared to unaffected individuals [3]. Whereas patients with OCA1A generally have white hair and eyelashes, pale skin and translucent irides, patients with OCA1B (previously called “yellow albinism”), have variable amounts of melanin pigment, which can increase over time. A subset of patients with OCA1B carry alleles for a temperature-sensitive form of tyrosinase with activity optima <37°C; as a result, pigment is generally more prominent in the extremities, where the temperature may be cooler than in other parts of the body [4], [5].

Temperature sensitive albinism is rare and is associated with a particular missense mutation in the tyrosinase gene [6]. The mutation, R422Q, results in a temperature sensitive trafficking defect preventing the translocation of the mutant tyrosinase into endosomes when expressed in COS7 cells [7]. At 37°C, mutant R422Q tyrosinase is retained in the endoplasmic reticulum and is possibly degraded by proteasomes with no pigment production. In contrast, in pigmented tissues at lower temperatures (31°C) the enzyme is translocated into the endosomes where it produces pigment. This leads to a phenotype reminiscent of the Siamese cat with no pigment centrally but pigmentation develops in the extremities (ears, face, legs and tail) [8]. A tyrosinase missense substitution H420R has been observed in the Himalayan mouse [9]. Both the above-mentioned murine and human tyrosinase substitutions occur within a very highly conserved segment of the protein, and it is likely that they result in similar instabilities of the corresponding tyrosinase polypeptides.

Tyrosinase catalyze the first two steps of the melanin synthesis pathway, hydroxylation of L-tyrosine to L- 3, 4-dihydroxyphenylalanine (L-DOPA, monophenolase or cresolase activity, EC1.14.18.1)) and the subsequent oxidation of L- DOPA to dopaquinone (diphenol oxidase or catecholase activity, EC 1.10.3.1) [10]. Tyrosinase also catalyzes the subsequent oxidation of 5,6-dihydroxyindole and 5,6-dihydroxyindole-2-carboxylic acid into indole-5,6- quinone and indole-5,6-quinone carboxylic acid, respectively. Human tyrosinase is found specifically in neural crest-derived pigment-producing cells (melanocytes) of the skin, choroid and iris and in the neuroectoderm-derived RPE of the eye [11]. Tyrosinase has been isolated from bovine eyes [12] and melanoma cells [13], [14], and the full-length human tyrosinase gene cloned [15]–[17].

The atomic structure of the human tyrosinase has not been determined, but based on the sequence of the enzyme, is predicted to contain several functional domains: an epidermial growth factor (EGF)-like domain, an enzyme’s catalytic domain which has a structure similar to that of mushroom tyrosinase [18], [19], and a trans-membrane (TM) domain (http://www.uniprot.org/uniprot/P14679). Although the functional role of the EGF-like domain is unknown, monophenolase and diphenol oxidase activities are associated with the tyrosinase enzymatic domain which contains two copper ions coordinated by 6 histidine residues at the catalytic site [1]. The EGF-like and tyrosinase enzymatic domains are part of a globular domain anchored by the adjacent trans-membrane domain and extending into the vacuolar compartment of melanosomes, the intracellular organelle that compartmentalizes melanin [20].

To better understand protein function and the pathogenic effects of genetic mutations on human tyrosinase, we expressed the human intra-melanosomal domain of wild-type and temperature-sensitive mutant tyrosinase in whole insect *Trichoplusia ni (T. ni)*. Purified wild-type and mutant proteins were glycosylated and enzymatically active. The nature of the temperature sensitivity of the mutants was examined by conformational and activity analyses under various conditions.

## Results

### Human Tyrosinase hTyrC_tr_ Expression, Purification, and Characterization

#### Recombinant truncated hTyrCtr is a soluble protein

**Figure 1:**
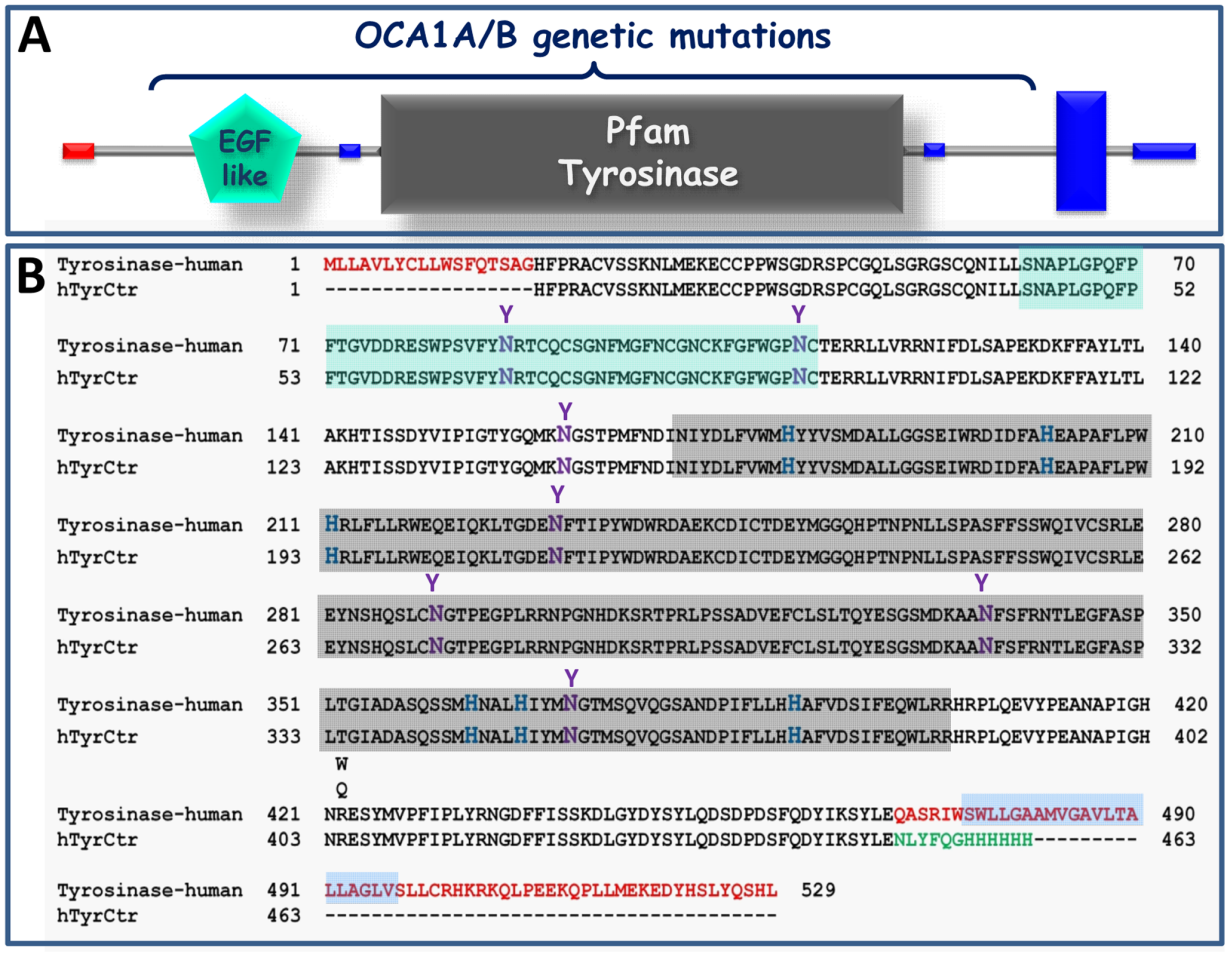
Recombinant protein hTyrC_tr_ consists of the intra-melanosomal portion of human tyrosinase. **A**: The domain structure of human tyrosinase was obtained using the SMART (http://smart.embl-heidelberg.de). Red = signal peptide; green = EGF-like domain; gray = catalytic (tyrosinase) domain; blue = trans-membrane domain. **B**: The alignment of human tyrosinase and human tyrosinase intra-melanosomal domain sequences. The intra-melanosomal domain is located between the N- terminal signal peptide and a truncated C-terminus. The 18 N- and 70- C-terminal residues of the boundary are shown in red. A TEV cleavage site and a 6His-Tag are shown in green. Potential N-glycosylation sites, N86, N111, N161, N230, N290, N337, and N371, and copper-binding sites coordinated by His residues H180, H202, and H211 (A-site) and H363, H367, and H390 (B-site), are shown in magenta and blue, respectively. Locations of two temperature-sensitive mutations R422Q/W are shown. The sequence molecular mass of hTyrC_tr_ is 53.129 kDa and the calculated pI is 5.70. Sequence fragments related to protein domains are shown by background colors: light green (EGF-like); light grey (tyrosinase) and light blue (trans-membrane helix).

The human tyrosinase is a Type I membrane glycoprotein composed of several predicted functional domains (Figure 1A: an 18 amino acids signal sequence at the amino-terminus, an EGF-like domain (residues 60–112), a catalytic domain (residues 170–403), and a C-terminal trans-membrane helix domain (residues 474–496)), as suggested by SMART (http://smart.embl-heidelberg.de). Expression and purification of membrane proteins can be problematic, so we truncated the C-terminal transmembrane domain and expressed the intra-melanosomal portion of human tyrosinase, hTyrC_tr_ (residues 19–469) as a C-terminal hexa-histidine fusion protein. To date, 295 unique sequence variations in the tyrosinase have been reported in OCA1 patients (Human Gene Mutation Database, HGMD professional, ver. 2011.4 (http://www.hgmd.org/). Within this dataset, 233 missense/nonsense variants were found, greater than 90% of which are in residues 1–460. Hence, the vast majority of human disease mutations can be tested by expressing only the intramelanosomal fragment of tyrosinase (Figure 1B).

**Figure 2:**
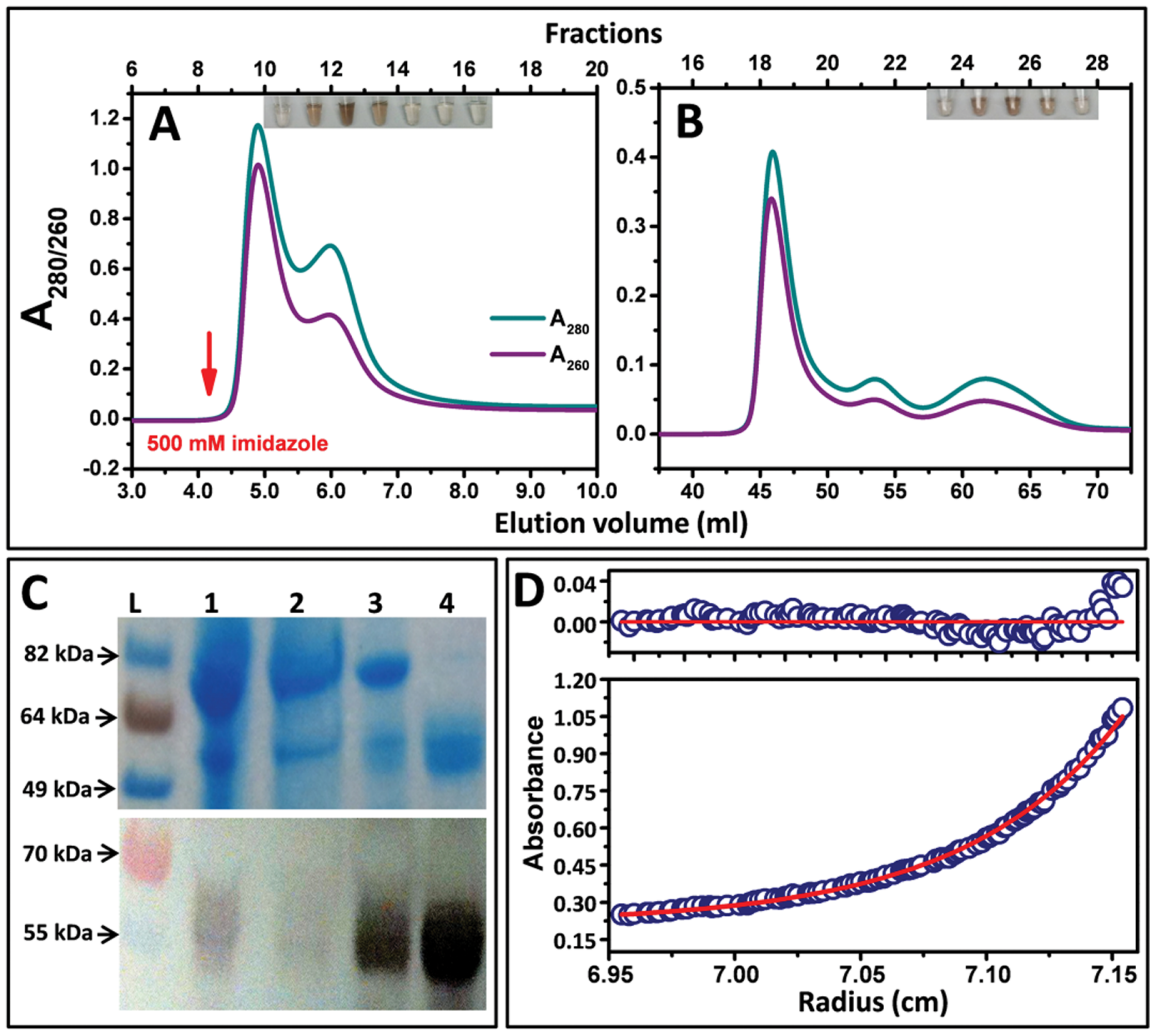
Purification and characterization of hTyrC_tr_. **A**: IMAC using a HisTrap 5 ml column. The arrow indicates the start of the imidazole gradient. **B**: Gel filtration using Superdex 75 16/60 HR. Chromatography profile monitored at 260 nm (purple lines) and 280 nm (green lines). The inserts show the diphenol oxidase activity of hTyrC_tr_ measured in separate tube for each fraction after 30 min of incubation at 37°C with 3 mM L-DOPA in 50 mM sodium phosphate buffer, pH 7.5. **C**: SDS-PAGE (top panel) and Western blot (bottom panel) showing stepwise purification of hTyrC_tr_. From left to right: L, protein ladder; 1, total lysate of larvae expressing hTyrC_tr_; 2, flow through; 3, sample after 5 ml HisTrap; 4, sample after Superdex 75. **D**: Sedimentation equilibrium of hTyrC_tr_. The protein concentration gradient (280 nm) versus radial distance is indicated. The red line shows calculated fit for an ideal monomer and blue circles the experimental values. The top panel shows the residuals of a fitted curve to the data points. Calculated fit of monomer was obtained assuming that the average partial specific volume of glycans is 0.63 cc/g and that the protein contains 10% carbohydrate.

**Figure 3:**
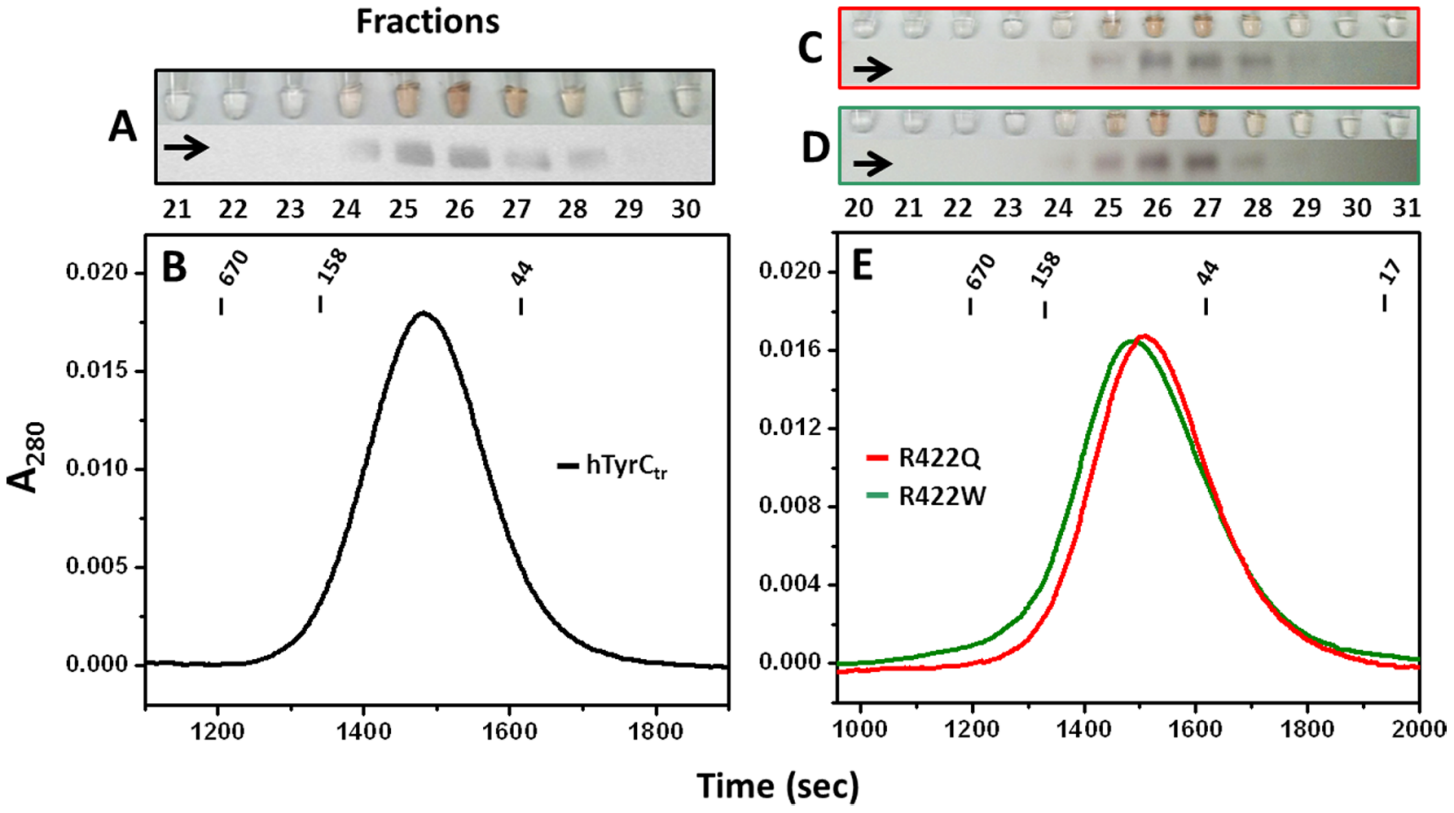
Gel filtration and enzymatic activities for the pure recombinant hTyrC_tr_ and two temperature sensitive mutant variants. Chromatographies were using Superdex 75 10/30 HR: hTyrC_tr_ (**B**) and of R422Q and R422W (**E**). The elution points of molecular mass standards are shown at the top for reference. Panels **A, C** and **D** show test tubes containing the L-DOPA colorimetric reactions for each protein fraction of hTyrC_tr_, R422Q and R422W, respectively. Brown color (intensity proportional) in tube indicates diphenol oxidase activity. Corresponding Western blots bands were labeled by horizontal arrows.

Recombinant hTyrC_tr_ was expressed in whole insect *T. ni* larvae. Western blotting of tyrosinase on larval extracts showed bands with molecular mass ∼ 52–64 kDa for wild-type hTyrC_tr_ (Figure S1A, Lane 3). This molecular weight heterogeneity is due to glycosylation (e.g., N-linked glycosylation) of the enzyme as described below. Proteins were purified by immobilized-metal affinity and size-exclusion chromatography (IMAC and SEC, respectively) as shown in Figure 2A–B. Initially, the purification steps were monitored by SDS-PAGE and Western blotting analyses (data not shown). However, we found that hTyrC_tr_ fractions could be localized in each step by a simple assay, using a colorimetric reaction with L-DOPA (Figure 2A–B, inserts). Brown color in test tubes indicates fractions with tyrosinase activity: fractions 12–14 (Panel A) and fractions 25–27 (Panel B). SDS-PAGE and Western blot analyses (Figure 2C) indicate the purity of recombinant tyrosinase polypeptide at each stage of purification. The purified protein ran as a broad band on SDS-PAGE 52–64 kDa (Figure 2C, lane 4) and eluted from a gel filtration column in a single peak with ∼ 57 kDa molecular mass (Figure 3B). hTyrC_tr_ purification is summarized in Table 1 with mass balances indicated. The relative purity was ∼ 1.6 mg of pure hTyrC_tr_ from 10 g of larval biomass.

**Table 1:** Purification of recombinant hTyrC_tr_ and two temperature sensitive variants from 10 g of larval biomass^a^.

	Total protein (mg)		
Purification step	hTyrCtr	R422Q	R422W
**Lysate**	1668	1574	1816
**Affinity**	18.34	14.33	15.28
**SEC**	1.59	0.85	1.3
	**Specific L-DOPA activity (U/mg)**		
**Lysate**	4052	1850	1929
**Affinity**	20769	8681	12211
**SEC**	68207	52593	62391
	**Relative purity (%)**		
**Lysate**	4.1	6.1	5.5
**Affinity**	15	8.5	10.6
**SEC**	96.1	94.7	95.2

^a^ The total protein was estimated by the Warburg – Christian method using absorbance at 260/280 nm (Sigma-Aldrich); L-DOPA activity was determined as described in Materials and Methods section. Specific activity was obtained as the L-DOPA enzyme activity multiplied by the sample total volume and divided by total protein. The tyrosinase content of hTyrCtr, R422Q and R422W in protein extracts were estimated from SDS-PAGE gels as 100% X tyrosinase band intensity/total protein band intensity.

#### Recombinant hTyrCtr enzymatic activity

**Figure 4:**
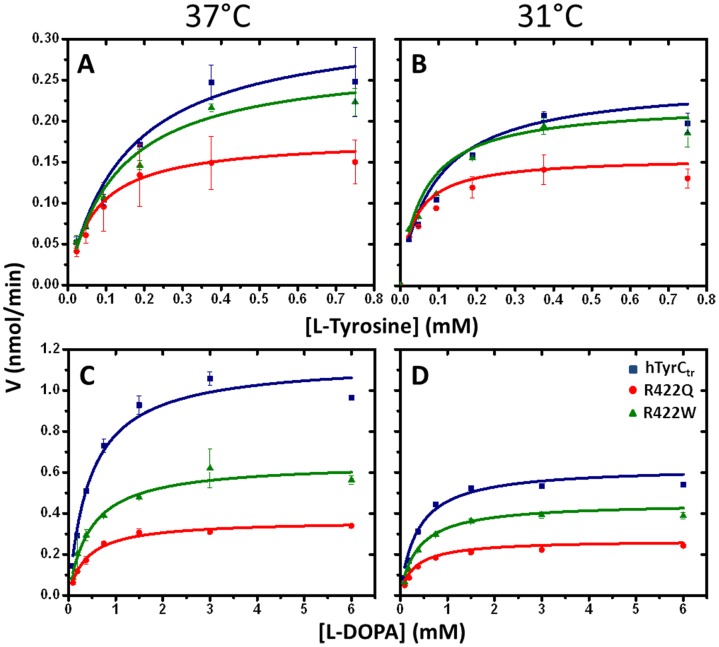
Kinetic analysis of hTyrC_tr_ and two mutants. Michaelis-Menten plots of monophenolase (**A**, **B**) and diphenol oxidase (**C**, **D**) activities of hTyrC_tr_ (blue) and two mutants, R422Q (red) and R422W (green), as a function of L-tyrosine and L-DOPA concentrations. Enzyme assays were measured at 37°C (**A**, **C**) and 31°C (**B**, **D**). The lines represent nonlinear fits to the Michaelis-Menten equation. Experiments were performed in triplicate and error bars represent standard deviations.

The enzymatic activities of recombinant hTyrC_tr_ were tested in *in vitro* with the substrates and inhibitors known to interact with mammalian tyrosinases (http://www.brenda-enzymes.info). The Michaelis-Menten constant (*K*_m_) and maximal velocity (*V*_max_) of proteins were calculated from Michaelis-Menten plots shown in Figure 4 (blue). Recombinant hTyrC_tr_ demonstrated two catalytic activities: monophenolase (*K*_m_ = 0.16±0.04 mM) and diphenol oxidase (*K*_m_ = 0.46±0.09 mM), with tyrosine and L-DOPA as substrates, respectively (Figure 4A, C and Table 2). The optimum temperature for both monophenolase and diphenol oxidase activities was 37°C (Figure S2A, C). Monophenolase activity was higher than diphenol oxidase over the pH range, 7–9; diphenol oxidase activity had a maximum at pH 7.5 (Figure S2B, D). Both monophenolase and diphenolase activities were inhibited by well-known inhibitors such as kojic acid (IC_50_ 117,185 µM), NaCl (IC_50_ 168, 225 mM), hydroquinone β-D-glucopyranoside (arbutin, IC_50_ 7.5, 7.8 mM ) and were 100% inhibited by the reducing agents dithiothreitol (DTT, 0.2 mM) and β-mercaptoethanol (β-ME, 0.01 mM). Finally, both activities were stimulated by HAA (Figure S3). These results confirm that removal of the membrane associating C-terminal domain did not affect activity of hTyrC_tr_, which was similar to that of observed for other tyrosinases (Table 3).

**Table 2:** Kinetic parameters for tyrosinase-catalyzed reactions.

Monophenolase activity				
37°C				
	K_m_ mM	V_max_ nmole/min	k_cat_ min^−1^	k_cat_/K_m_ mM^−1^ min^−1^
**hTyrCtr**	0.16±0.04	0.32±0.03	0.68±0.06	4.25±0.40
**R422Q**	0.07±0.01	0.17±0.01	0. 36±0.02	5.14±0.34
**R422W**	0.13±0.03	0.27±0.02	0.57±0.04	4.38±0.29
**31°C**				
**hTyrCtr**	0.10±0.02	0.24±0.02	0.51±0.04	5.10±0.34
**R422Q**	0.04±0.01	0.15±0.01	0.32±0.02	8.00±0.53
**R422W**	0.07±0.02	0.21±0.01	0.45±0.02	6.43±0.31
**Diphenol oxidase activity**				
**37°C**				
**hTyrCtr**	0. 46±0.09	1.15±0.06	11.45±0.59	24.89±1.28
**R422Q**	0.39±0.04	0.36±0.01	3.61±0.09	9.26±0.23
**R422W**	0.46±0.08	0.64±0.03	6.42±0.30	13.96±0.65
**31°C**				
**hTyrCtr**	0.38±0.07	0.62±0.03	6.21±0.30	16.34±0.79
**R422Q**	0.33±0.03	0.25±0.01	2.50±0.05	7.58±0.15
**R422W**	0.39±0.05	0.44±0.02	4.4±0.28	11.28±0.71

Here *K*_m_, Michaelis-Menten constant defines the affinity of the substrate for the enzyme; *V*_max_, the rate at which a substrate will be converted to product once bound to the enzyme; *k*_cat_ the enzyme turnover, number of substrate molecules turned over per enzyme molecule per minute. It is defined to equal *V*_max_/*E*_t_, where *E*_t_ is concentration of enzyme in µmol; *k*_cat_/*K*_m_, enzyme efficiency.

**Table 3:** Effect of Inhibitors on enzymatic activity of hTyrC_tr_ is shown by IC_50_ values.

	IC-50 values		
Inhibitor	Monophenolase activity, *hTyrC_tr_*	Diphenol oxidase activity, *hTyrC_tr_*	Reported
**Kojic acid**	117 µM	185 µM	7.4–700 µM
**NaCl**	168 mM	225 mM	0.5–360 mM
**Arbutin**	7.5 mM	7.8 mM	0.04–548 mM
**DTT**	n/a	0.2 mM**	0.1–1 mM**
**β-ME**	n/a	0.01 mM**	0.1–2 mM**

Values for tyrosinase enzymes (EC 1.14.18.1) obtained from different sources and summarized in the BRENDA database, http://www.brenda-enzymes.info); ^**)^ complete inhibition.

#### Recombinant hTyrCtr is a monomeric glycoprotein

SEC and sedimentation equilibrium were used to measure the oligomeric state of hTyrC_tr_. Gel filtration of hTyrC_tr_ on a calibrated column (Figure 3B) indicates a molecular weight of ∼ 57 kDa. This was confirmed using sedimentation equilibrium (Figure 2D) where a *M*_r_ of 56,630 kDa was determined for the glycosylated protein. The centrifugation result was obtained assuming a 10% carbohydrate content as described in Table S1.

Human tyrosinase is modified post-translationally by N-linked glycosylation on asparagine (Asn, N) residues; this glycosylation may be important for enzymatic activity [21]. To study the glycosylation pattern of the recombinant enzyme, hTyrC_tr_ was treated with peptide-N4-(N-acetyl-beta-glucosaminyl) asparagine amidase (PNGase F) to remove Asn-linked glycans. SDS-PAGE of the deglycosylation protein indicates a single protein band with molecular mass of ∼55 kDa (Figure S1C). The PNGase treatment removes the band heterogeneity and shows that hTyrC_tr_ is N-glycosylated, similar to enzyme purified from cell culture [22].

Based on the gene sequence, hTyrC_tr_ is predicted to have 28 Asn residues, of which 7 (N-86, -111, -161, -230, -290, -337, and -371) have the N-xxx-S/T sequence motif for potential N-glycosylation. Five of the Asn residues (N-86, -111, -230, -337, and -371) are predicted to have a high probability (50–75%) for glycosylation using NetNGlyc 1.0 (http://www.cbs.dtu.dk/services/NetNGlyc/).

Localization of the N-glycosylation sites and the extent of modification for each site were determined by enzymatic removal of the N-linked carbohydrates with PNGase F followed by tryptic mapping. PNGase F hydrolyzes Asn-linked carbohydrates, resulting in deamidation of the modified Asn residue linking the carbohydrate, converting it to an Asp residue. The deamidated Asn can be detected by an increase of 1 Da, corresponding to the conversion of the -NH_2_ to -OH. The results of LC/MS/MS data from tryptic digests of hTyrC_tr_, collected using a Q-TOF mass spectrometer, are summarized in Tables S2A, B. The peptides containing potential N-glycosylation sites were matched to the observed MS/MS spectra for PNGase treated hTyrC_tr_. Tryptic peptides containing N86 and N337 were not observed with untreated protein (control) while the corresponding deamidated peptides were present after PNGase F treatment, indicating that both Asn’s were fully glycosylated. In contrast, unmodified peptides containing N-111, -230, and -290 were present in digests of the control indicating only partial glycosylation of these sites. After PNGase F treatment, the relative levels of deamidated peptides increased, and the ratios of deamidated to Asn containing peptides indicated that these sites were 40–60% glycosylated. For the peptide containing N290, partial deamidation of Asn was observed in the control; this may be due to spontaneous deamidation as Asn-Gly dipeptide sequences, such as at N290, have high rates of spontaneous deamidation [23]. The level of deamidated N290 peptide ions increased after treatment with PNGase F, indicating partial carbohydrate modification; however, the level of modification could not be accurately determined.

#### Site-specific N-linked carbohydrates

Glycopeptide spectra were identified by comparison of LC/MS/MS parent ion peak lists for ions present in PNGase F treated and control samples. The fragmentation spectra for these candidate ions were then examined for carbohydrate-specific oxonium ions of m/z 204, 292, and 366. The mass lists of these parent ions were entered into GlycoMod with the predicted hTyrC_tr_ amino acid sequence to identify potential combinations of tryptic peptides and carbohydrates to produce the observed mass. Ten parent ions, corresponding to eight specific glycopeptides were identified. The results are shown in Tables S2A, B.

Two of the Asn-sites (N111 and N337) were found to be modified by the same carbohydrate composition (Hex_2_HexNAc_2_Deoxyhexose_1_) corresponding to a 876.322 Da (monoiso) residue mass. Some N93 was modified by only a single HexNAc residue (HexNAc, N-acetyl hexoseamine; Table S3). Fragmentation spectra from the modified peptides indicate that a HexNAc residue is directly linked to the peptide (peptide+HexNAc ion), and deoxyhexose is also linked to this first HexNAc residue (peptide+HexNAc+deoxyhexose ion). Membrane glycoproteins from *Bombyx mori* have been shown to be modified by N-linked carbohydrates with the same composition, which was found to have the following structure, Man(a1-6)Man(b1-4)GlcNAc(b1-4)[Fuc(a1-6)]GlcNAc (Man, mannose; GlcNAc, N-acetylglucoseamine; Fuc, fucose); [24]. As hTyrC_tr_ was expressed in an insect larvae system, it seems likely that the same carbohydrate structures are linked to N111 and N337 of hTyrC_tr_.

**Figure 5:**
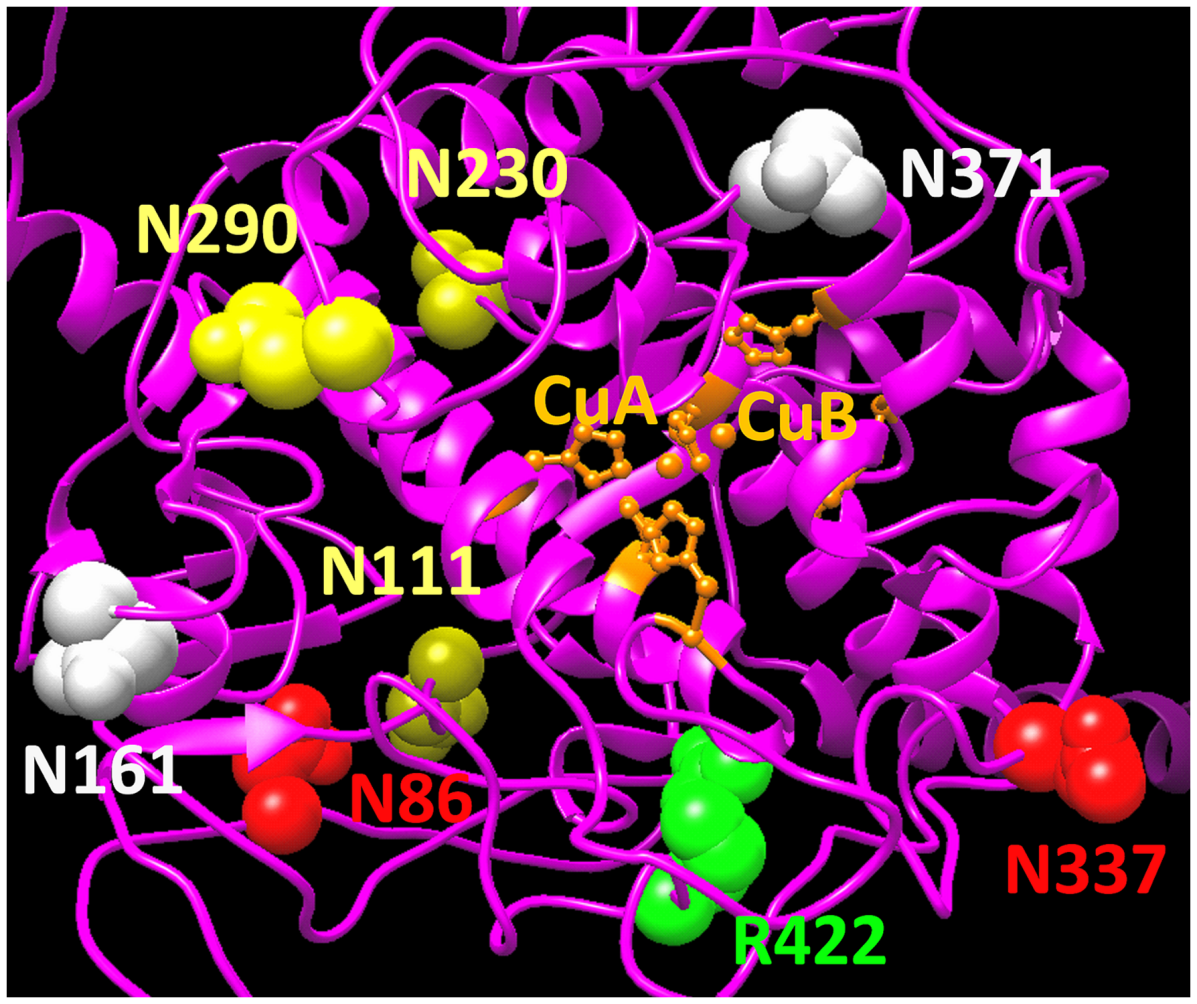
N-glycosylation of hTyrC_tr_. N-glycosylation sites determined by MS are mapped to the human tyrosinase protein structure modeled as described in methods section. The protein backbone structure is shown by magenta ribbon. Two copper atoms, CuA and CuB, which coordinated by His residues are shown in orange. Fully and partially occupied N-glycosylation sites are represented by red and yellow spheres, respectively. Two potential N-glycosylation sites, not determined in in present study, are shown in grey. The location of mutant variants is indicated in green.

**Figure 6:**
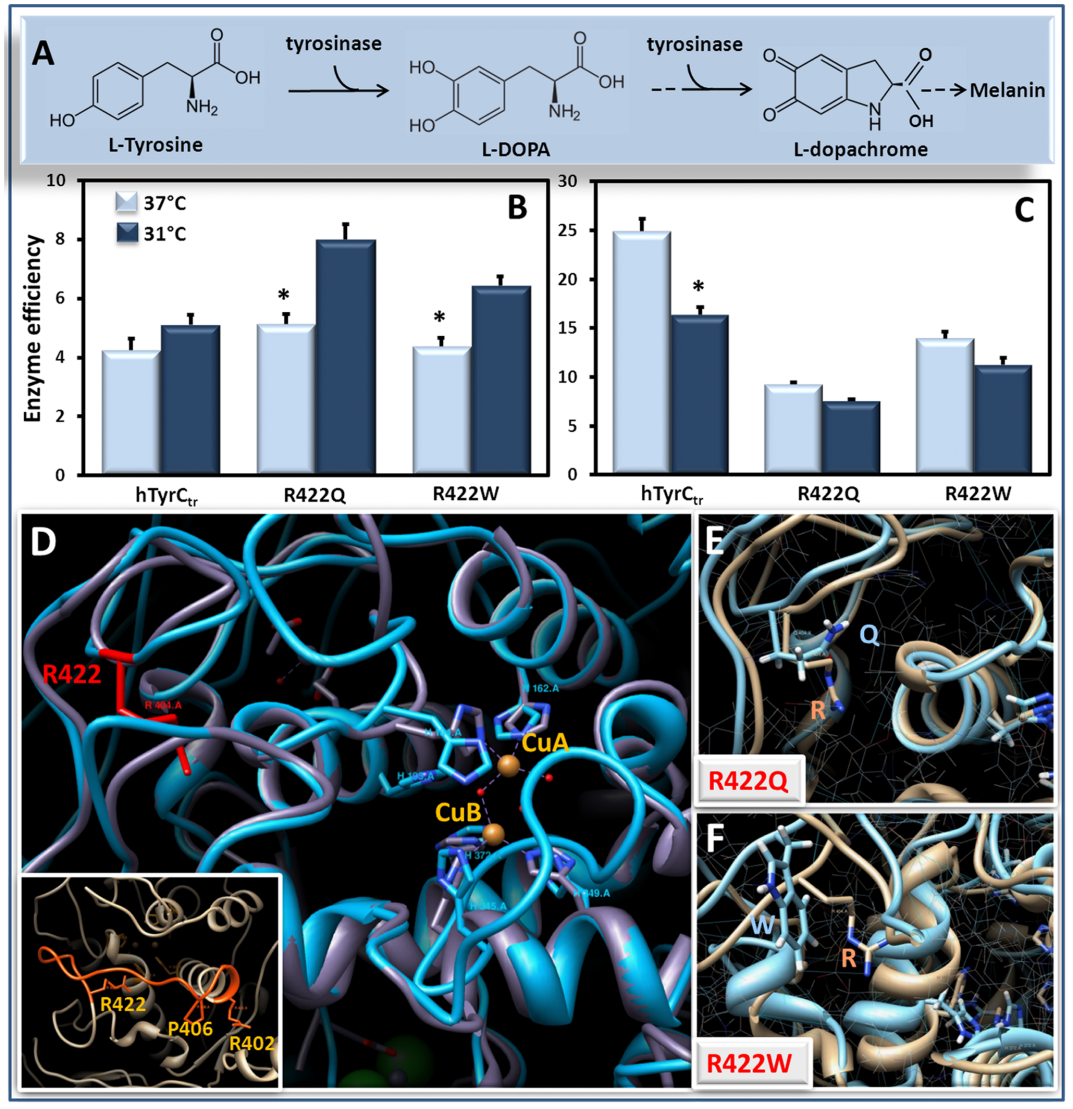
Catalytic efficiencies of hTyrC_tr_ and mutants R422Q and R422W as a function of temperature. Panel **A** shows a schematic view of first two steps of the melanin biosynthesis pathway regulated by tyrosinase. The catalytic efficiency (k_cat_/K_m_, mM^−1^ min^−1^) of the monophenolase (**B**) and diphenol oxidase (**C**) activity obtained from the Table 2 for hTyrC_tr_ and mutant variants R422Q and R422W at 37 and 31°C are shown by light blue and dark blue bars, respectively; *p<0.05. Protein structure of the wild type hTyrC_tr_ and temperature-sensitive mutant variants, R422Q and R422W are shown on Panels **D, E** and **F**, respectively. Structural superposition of human tyrosinase hTyrC_tr_ and the bacterial tyrosinase (PDB file: 3nm8) shown by grey and cyan, respectively. Temperature-sensitive mutations at positions 402, 406 and 422 are located in the same structural fragment shown by orange.

In summary, hTyrC_tr_ contain five N-glycosylation sites which are either completely modified (N86, N337) or partially modified (N111, N230, N290). Molecular modeling of human tyrosinase suggests that these N-glycosylation sites are located at the protein surface (Figure 5) and do not affect directly the 4-helix bundle structure maintaining the copper-binding sites (Figure 6D).

### Temperature-sensitive Mutant Variants R422Q and R422W

#### Protein expression/purification

Some patients with OCA1 carry missense alleles that behave in a temperature-sensitive manner where residual tyrosinase activity is detected at 31°C, but is nearly abolished at physiological temperature (37°C) [22]. These mutations are of particular interest because at least one example appears to be amenable to pharmacological stimulation in a mouse model [25]. Further, a partially-functioning enzyme would be a useful tool in a high-throughput drug screen for other stimulatory compounds. To examine the effect of temperature sensitive mutations at the molecular level, two variants, R422Q and R422W, were produced using the same methods as for hTyrC_tr_. The purification of two mutant variants is summarized in Table 1. Gel filtration of the R422Q and R422W mutants indicated the proteins were monomeric with estimated molecular masses of 55 and 56 kDa, respectively (Figure 3E).

Both variants had lower L-DOPA activities compare to wild type protein (Figure S4A). Following endoglycosidase F1 treatment under native conditions, the activities of wild type protein and two variants showed little change. Western blotting of the variants shows bands with molecular masses similar to that of wild type protein both before and after deglycosylation (Figure S4B). In summary, hTyrC_tr_ is N-glycosylated: mutant variants are glycoproteins which are monomeric and have masses very similar to wild type protein (Figure 3C–E and Figure S4B).

#### Enzymatic activity

Previous studies have shown that temperature sensitive mutants exit the endoplasmic reticulum and enter the endosomal compartment at 31°C, but not 37°C, suggesting that intracellular targeting is an important mechanism for loss of enzymatic activity [7], [26]. It is unknown however, whether such mutations *intrinsically* affect enzymatic activity and so we measured the temperature and pH dependences of catalytic activity of the wild-type and mutant proteins at 31 and 37°C (Figure S2, Table 2, and Figure 4). All enzymes have optimum activities at about 37°C but overall the mutant variants were less active than the wild-type enzyme. With the diphenol oxidase assay, the pH optima of wild type and R422W mutant were pH 7.5, whereas, the R422Q mutant showed very low activity change over the pH range studied. With the mutants, direct measurements of catalytic activity (Figure 4, Figure S2) did not reveal difference in enzyme activity between 31 and 37°C, however; this is not true when catalytic efficiencies (*k_cat_/K_m_*) are compared. For example, the monophenolase activities of both mutants have lower efficiencies at 37°C compared to 31°C, whereas the wild–type protein does not show this effect (Figure 6B, Table 2). The temperature sensitivity of the mutants could be due to substrate binding with higher affinity at 31°C compared to 37°C (Table 2). For diphenol oxidase activity, both mutants showed no temperature effect, however; both had lower efficiencies compared to wild-type enzyme (Figure 6C). Overall the results indicate that the two mutants exhibit dissociated temperature sensitivity in that the first rate limiting step in tyrosine conversion (monophenolase oxidase) is sensitive but the second step (diphenol oxidase) is not.

#### Circular dichroism (CD)

**Figure 7:**
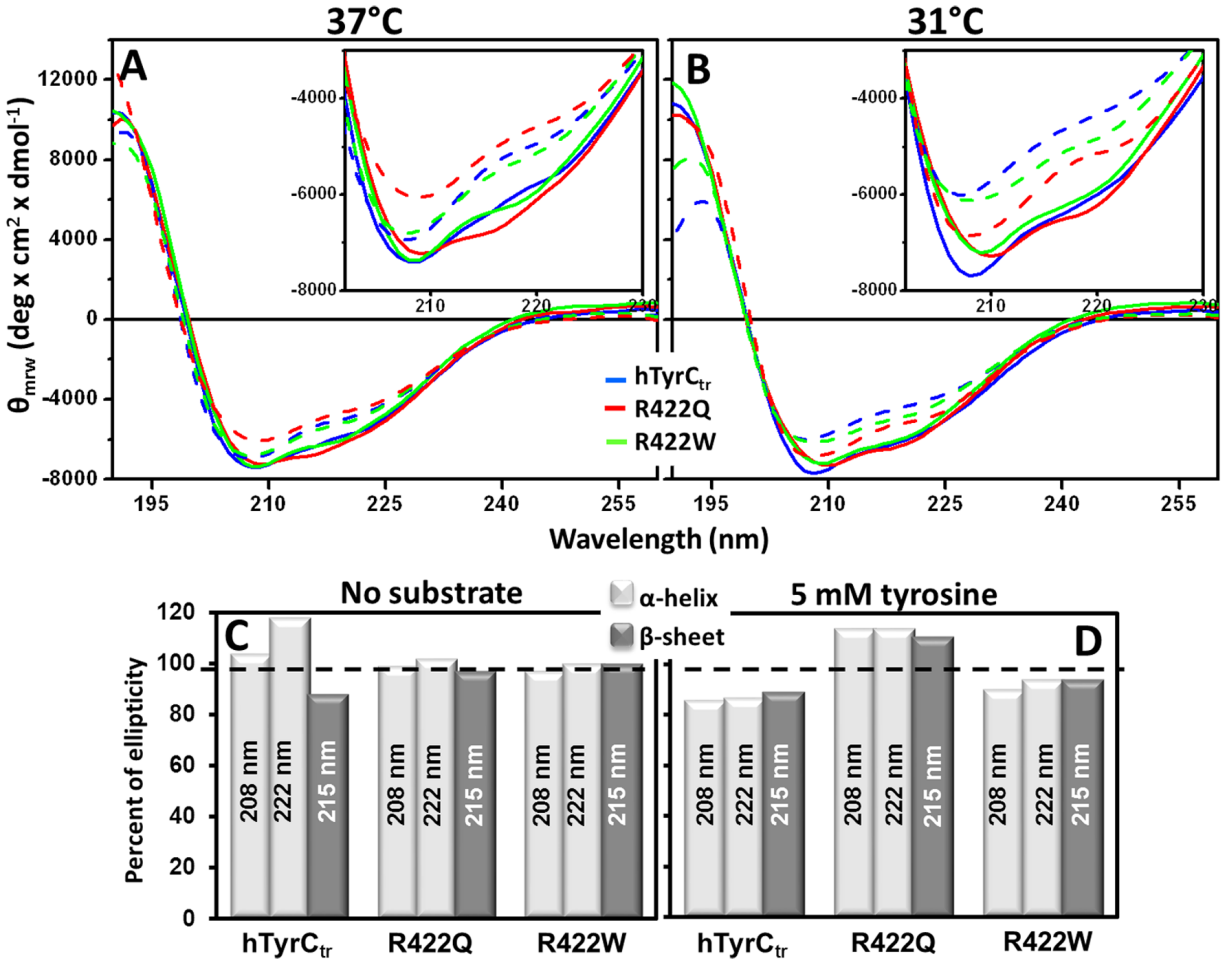
Far-UV CD Spectra of hTyrC_tr_ and temperature sensitive mutants R422Q/W. CD spectra for hTyrC_tr_ and two mutants, R422Q and R422W, are shown by blue, red, and green solid lines, respectively. Dashed lines show correspondent spectra measured in the presence of 0.5 mM tyrosine. Measurements were performed at 37°C (**A**) and 31°C (**B**). Scans (190–260 nm) were performed in 50 mM sodium phosphate buffer, pH 7.5 at protein concentrations of 0.2 mg/ml. **Inserts**: spectral differences at the range 200–230 nm are shown. Histograms C and D show ellipticity (Θ) ratios (%) in the absence (**C**) or the presence (**D**) of tyrosine with data from spectra shown in Panels **A**, **B**. The ratios were calculated as 100% × (Θ) 31°C/(Θ) 37°C determined at fixed wavelengths of 208, 222 nm (α-helix), and 215 nm (β-sheet). Dashes show a 100% level.

To examine if the temperature-sensitive changes in catalytic efficiency were due to structural perturbations we monitored conformation using far-UV CD. In the absence of substrate, the spectra of hTyrC_tr_ and the mutants were similar at 31 and 37°C suggesting little or no change in secondary structure (Figure 7A–B, solid lines).

In the presence of substrate; however, there was a significant difference between the spectra of hTyrC_tr_ and the mutant R422Q at 31 and 37°C (Figure 7A–B). In contrast, the R422W mutant showed little change. These spectral shifts reflect structural changes and deconvolution of the CD spectra was used to identify which secondary structure elements were involved (Figure S5). Hence, compared to WT enzyme, the R422Q mutant at 37°C had lowered helical and increased β-sheet content. This conformational change is equivalent to partial (localized) protein unfolding with concomitant reduction in the enzyme’s function. Hence, temperature dependent changes in catalytic efficiency of R422Q appear to be associated with structural change. The temperature sensitivity of the R422W mutant is not clearly detected by far-UV CD. More conformation sensitive methods and future structural studies will address this issue.

## Discussion

In this work we designed, expressed and purified the intra-melanosomal domain of human tyrosinase, hTyrC_tr_, and two temperature-sensitive mutants, R422Q and R422W, found in some patients with OCA1B. The recombinant proteins were modified by removing the C-terminal trans-membrane domain to avoid potential protein insolubility but retain all other functional features of the enzyme which reside in the remaining N-terminal sequence. Recombinant proteins (as hexa-histidine fusions) were produced in whole insect *T. ni* and purified by IMAC and SEC as active enzymes. After two chromatography steps the protein yield was >1 mg of purified protein per 10 g of larval biomass. The purified enzyme was a monomeric glycoprotein 57 kDa protein with maximum activity at neutral pH and 37°C. Glycosylation occurred at several Asn residues, and the enzymes’ kinetic and pharmacologic properties were similar to the authentic enzyme described in the literature [27]–[29]. We furthermore characterized the enzymology of two temperature-sensitive enzymes and showed for the first time that, at least in part, the temperature sensitive behavior is intrinsic to the protein structure.

Tyrosinase from lower organisms such as mushroom can be purified to homogeneity with low yield and is commercially available (ENZO Life Sciences, NY). Similarly, mammalian tyrosinase has been purified from various sources such as pigmenting melanoma cells [28]. Purification of the authentic enzyme is, however, complex and results in heterogeneous products with varying degrees of solubility. These purifications are usually not scalable and unlike recombinant produced material, mutations cannot be introduced to study the biochemistry of disease-related proteins. More recently, Kong et al. have reported expression of first 455 amino acid residues of human tyrosinase, the so-called ectodomain, in *E. coli* [30]. As expected from expression in a prokaryote, this soluble form of tyrosinase is not glycosylated and the purification yield was low. While this recombinant protein exhibited both tyrosine hydroxylation and oxidation activities, its biochemical properties were different from those reported for other mammalian enzymes including a high temperature maximum for activity (∼65°C) and a lower specific activity. Our attempts to produce active intra-melanosomal domain, hTyrC_tr_, in *E. coli* failed probably due to protein misfolding (data not shown). Switching to the baculovirus system resulted in the production of soluble expressed protein. The purified hTyrC_tr_ is enzymatically active and has biochemical properties similar to the published data, is post-translationally modified, and is optimally active at 37°C and neutral pH (Figure S2, blue line).

Tyrosinase undergoes highly-regulated and complex processing in the endoplasmic reticulum (ER) and Golgi apparatus on the way to its final destination in the melanosome, a specialized endosomal organelle. Bhatnagar et al. found that melanosomes purified from a mouse melanoma cell line had an internal acidic pH of ∼4.6 [31]. Similarly, Puri et al. found that C57BL6 mouse melanosomes were acidic but that the mutation in *OCA2,* a cause of albinism, resulted in isolated vesicles now having an internal neutral pH [32]. Counter to these findings, Ancans et al. reported that melanin synthesis occurred at neutral pH and proposed an alternative explanation for the effect of the OCA2 mutation [33], [34]. These conflicting results may not, however, be mutually exclusive of one another. Our in-vitro data indicates that although the pH optimum of human tyrosinase is 7.4 it is still active below pH 6.0 (Figure S2B, D) and, thus, in-vivo would be expected to function under potential sub-optimal conditions in the melanosome. Hence, the pH activity profile and pH optimum of purified enzyme are consistent with in-vivo activity under the physiological extremes reported in the literature.

Part of tyrosinase maturation is its post-translational N-glycosylation. Branza-Nichita and colleagues have reported that glycosylation is critical for tyrosinase folding, quality control and, therefore, activity [35]. Similarly, Imokawa et al. found that drugs that inhibit N-linked glycosylation inhibit tyrosinase and melanin production in melanoma cell lines [36]. These data are consistent with our molecular modeling, which shows glycosylated residues are located on the surface of the intra-melanosomal portion of tyrosinase, and so are accessible for interactions with other melanosomal proteins (Figure 5).

Glycosylation may, in fact, serve as a control point in regulating cellular tyrosinase activity. Ujvari et al. found that the rate of translation of tyrosinase, in part, determined its level of N-glycosylation [37]. Our purified enzyme is glycosylated at five of the seven potential sites of modification, although the extent on glycosylation at a specific residue may be variable. This variability may be an artifact due to the expression and purification or a natural maturation process. This is in contrast to published results using site-directed mutagenesis in melanoma cells [37], where all seven possible sites are modified. Of particular note, these authors found that N337 and N371 must be glycosylated for maximum enzyme activity; our MS analysis cannot identify glycosylation at N371. A study on glycosylation-deficient mutants suggested these two sites are critical for proper tyrosinase processing [38] and may be needed to preserve the active site conformation of the copper atoms at binding sites A and B [39]. In addition, the N371-glycosylation site is eliminated by N371Y or N371T mutations in individuals with OCA1 [40], [41]. Tyrosinase glycosylation may vary according to cell type and future studies will further address the role of N-glycosylation in folding, protein stability, and enzymatic activity.

Previous reports have shown that temperature-sensitive mutants of tyrosinase are targeted for degradation within the cell at 37°C, but undergo proper targeting at 31°C [42]. An important question is whether the temperature-dependent activity is intrinsic to the structure of the mutant tyrosinase *per se*. Both temperature-sensitive mutants, R422Q and R422W, show no significant difference for monophenolase and diphenol oxidase activities at 31 and 37°C. However, for the monophenolase reaction, the catalytic efficiency for both mutant variants was higher at 31°C with a significant decrease at 37°C (Figure 6, Panels A**–**C). Similar results have been reported for monophenolase reaction temperature-sensitive mutants [22] which are active at 31°C but essentially inactive at 37°C. Furthermore, our CD data (Figure 7) suggest that the temperature changes are associated with differences in the R422Q secondary structure. These results support the view that the temperature-sensitive reduction in catalytic efficiency, for at least one of the mutants, is directly related to structural change.

It is interesting that known temperature-sensitive mutations are found in positions 402, 406, and 422 (Figure 6D, insert) which localized on the same surface loop of the protein. This suggests that these mutations produce similar conformation changes, leading to a shared pathogenic mechanism. This could include an effect on the catalytic efficiency of the monophenolase activity, as observed in our model, as well as a common pathway of targeted degradation within the cell at the non-permissive temperature.

A major morbidity in albinism is reduced best-corrected visual acuity - including legal blindness - likely due to hypoplasia of the fovea, a highly specialized area of the neural retina [43]. While the precise relationship between the development of melanin pigment in the retinal pigment epithelium (RPE) and foveal development is not known, improving melanin production during postnatal retinal development has the potential to improve vision in patients with OCA1. We have recently reported that nitisinone, an FDA-approved drug, improves ocular and fur pigmentation in a mouse model of OCA1B [25] and are developing a pilot clinical trial to test its ability to improve pigment production in patients with OCA1B. A major hurdle in developing novel compounds that may stabilize tyrosinase and improve its ability to produce melanin is the lack of purified human enzyme.

In conclusion, we have described methods which result in the production of pure wild-type and mutant tyrosinase proteins in quantities sufficient for targeted or panel drug screening. Pure recombinant enzyme would also have the potential for direct therapeutic treatment, if delivered to the retinal pigment epithelium of the eye prior to foveal development. Purified proteins can also be used for crystal screening and X-ray structure determination. High resolution structural information could be used, for example, in rational drug designs. Also, structural determinations could help understand at a molecular level how individual mutations contribute to the diverse phenotype in patients with albinism.

## Materials and Methods

### Protein Expression and Purification

Recombinant human wild type tyrosinase, hTyrC_tr_, and R422Q and R422W mutant proteins associated with OCA1B were commercially produced in whole insect *T. ni* larvae (C-PERL Inc., MD). For all proteins, the 80 residues at the C-terminus comprising the trans-membrane domain were deleted to prevent potential protein insolubility. In addition, a TEV-protease cleavage site (NLYFQG sequence) and a 6His-Tag were added at the C-terminus of the truncated protein to facilitate protein purification (Figure 1B). However we did not perform TEV cleavage to remove the tag. To improve baculovirus expression, genes were codon optimized for the host insect *T. ni* and the native signal peptide (residues 1–21) was substituted with an insect signal sequence. Synthetic genes were cloned into baculovirus vector and co-transfected in Sf9 cells to generate baculovirus co-expressing the target gene with chaperones and helper proteins, in an effort to reduce proteolytic degradation and increase protein glycosylation. The resulting virus was injected into *T. ni* larvae and large-scale production was accomplished by oral infection using proprietary PERLXpressTM technology (C-PERL, Inc.).

hTyrC_tr_ was purified as follows. Infected larvae, frozen at −80°C, were disrupted by Omni Tissue Homogenizer using the Hard Tissue Omni Tip™ Homogenizer Probes (Omni International, GA) in 5 × (vol/weight) Buffer A: 20 mM sodium phosphate, 500 mM NaCl, and 20 mM imidazole, pH 7.4, in the presence of 25 µM 1-Phenyl-2-thiourea, PTU (Sigma-Aldrich, MO), 2 mM MgCl_2_, 40 µg/ml DNAse I (Thermo Fisher Scientific, PA), 0.2 mg/ml lysozyme and Complete set of protease inhibitors (Roche, USA). The homogenate was sonicated with an Ultrasonic Processor GE130PB (Hielscher System, Germany) with a 10 second pulse/10 second pause for 15 min. The lysates were centrifuged at 10,600×*g* for 30 min at 4°C and the supernatants diluted in 1∶1 ratio with Buffer A. Proteins were purified by IMAC and SEC at room temperature using a Bio-Logic Duo-Flow Maximizer workstation (Bio-Rad, CA). Soluble extracts were loaded on 5 ml His-Trap FF Crude IMAC column (GE Healthcare, NJ) equilibrated with Buffer A and eluted at flow rate of 1 ml/min. A gradient of 0–500 mM imidazole was applied and 2.5 ml fractions were collected. Fractions contained hTyrC_tr_ were identified using a color reaction to L-DOPA (see Tyrosinase enzymatic assays below). Active fractions were dialyzed overnight against 2 liters of Buffer B: 50 mM Tris-HCl, pH 7.2, 1 mM EDTA, and 150 mM NaCl, and concentrated into 5 ml. Proteins were further purified by SEC using 120 ml Superdex 75 16/60 and 30 ml Superdex-75 HR 10/30 columns (GE Healthcare, NJ), equilibrated with Buffer B at a flow rate of 0.5 ml/min; 2.5 and 0.5 ml fractions were collected, respectively. The columns were calibrated with protein standards (Bio-Rad, CA): thyroglobulin (670 kDa), γ-globulin (158 kDa), ovalbumin (44 kDa), myoglobin (17 kDa), and vitamin B12 (1.4 kDa). The void and total volumes of the column were determined with Blue Dextran 2000 and 50% acetone, respectively. Column fractions were monitored by *A*_280 nm/260 nm_ and by SDS-PAGE using 4–15% polyacrylamide gels (Bio-Rad, CA). Protein identity was confirmed by Western blot analysis using anti-tyrosinase T311 (Santa Cruz Biotechnology, CA) and anti-His (Life Technologies, NY).

### Tyrosinase Enzymatic Assays

Enzyme activities were determined according to a modified absorption assay [44], [45]. Protein concentrations were 0.25 mg/ml and 0.1 mg/ml for monophenolase and diphenolase activity experiments, respectively. Protein monophenolase and diphenol oxidase activities were measured using L-tyrosine and L-DOPA (Sigma-Aldrich, MO) as substrates, respectively. The reaction mixture containing 0.2 mM L-tyrosine or 3 mM L-DOPA in 50 mM sodium phosphate buffer, pH 7.5 was incubated for 30 min at 37°C and monitored by measuring the dopachrome formation at 490 nm (ε_dopachrome_ = 3520 M^−1^ cm^−1^) using a Varian Cary 300 Bio UV-Vis Spectrophotometer (Agilent Technologies, CA). For monophenol oxidase activity, additional 50 µM L-DOPA was added to eliminate a lag period [46].

### Kinetic Parameters

The monophenolase and diphenol oxidase reaction rates (*V*) were determined using the substrates L-tyrosine and L-DOPA at concentrations in the range of 0.023 to 0.75 mM and 0.094 to 6 mM, respectively. All assays were performed in 50 mM sodium phosphate buffer, pH 7.5 at 37°C and 31°C in triplicate. Absorbance was measured at 490 nm using VersaMax microplate reader (Molecular Devices, CA). The Michaelis-Menten constant (*K*_m_) and maximal velocity (*V*_max_) of proteins were calculated from Michaelis-Menten plots. Michaelis-Menten curves were fitted with corresponding nonlinear function using the OriginPRO program (version 9.0, OriginLAB Corporation, MA).

### Optimum Temperature and pH

Protein at 0.5 and 0.05 mg/ml with either 0.2 mM, L-tyrosine, or 1.5 mM, L-DOPA, were used for reaction mixtures. The mixtures were incubated for 30 min in 50 mM sodium phosphate and monophenolase and diphenol oxidase activities were determined at 490 nm at various reaction temperatures, 16–60°C (pH7.5), or over pH range 5.0–9.0 (37°C).

### PNGase F Deglycosylation

PNGase F digestion was according to manufacturer (Sigma-Aldrich, MO): hTyrC_tr_ was denatured in 50 mM sodium phosphate buffer, pH 7.5 by adding 0.2% SDS and 100 mM β-ME and heated at 100°C for 10 min. PNGase F (100 U) was added and the mixture was incubated at 37°C for 3 hours. Reactions were stopped by heating at 100°C for 5 min. Deglycosylated hTyrC_tr_ was analyzed by SDS-PAGE using 4–15% polyacrylamide gels (Bio-Rad, CA).

### Sedimentation Equilibrium

Analytical ultracentrifugation was carried out using a Beckman Optima XL-I analytical ultracentrifuge. Absorption optics, an An-60 Ti rotor and standard double-sector centerpiece cells were used. Equilibrium measurements at 20°C were made after 16–20 hours at 16,500 rpm. Baselines were established by over-speeding at 45,000 rpm for 4 hours. Data (the average of five scans collected using a radial step size of 0.001 cm) were analyzed using the standard Optima XL-I data analysis software. Protein partial specific volume was calculated from amino acid compositions [47]. Carbohydrate content was assumed to be 10% and using an average partial specific volume of 0.63 for N-linked carbohydrate [48], a partial specific volume of 0.711 g/mL was used for hTyrC_tr_.

### Circular Dichroism

A Jasco J-710 spectropolarimeter with 0.1 cm path-length cells were used. Scans 185–260 nm were performed in 50 mM sodium phosphate buffer, pH 7.5 with a protein concentration of 0.2 mg/ml. Spectra were measured in the presence and in the absence of 0.5 mM tyrosine salt. The reported CD spectra are the average of twelve scans corrected by subtraction of a buffer blank. Mean residue ellipticities were expressed for all wavelengths as deg×cm^2^×dmol^−1^ and were calculated from the equation *[θ] = θobs x M/10 x d x c*, where *θobs* is the measured ellipticity in degrees, M, the mean residue molecular weights (114.75, 114.69, and 114.82 for hTyrC_tr_, R422Q and R422W, respectively), *d* the optical path in cm and *c* the protein concentration in g/ml. Secondary structure parameters were estimated by the programs SELCON3 and CONTIN using DichroWEB [49].

### Carbohydrate MS Analysis

Recombinant hTyrC_tr_ protein (20 µg) was reduced with DTT and alkylated with iodoacetamide. Half of the sample was treated with PNGase F (2.5 units, Sigma, MO) for 2 hour at 37°C, and trypsin was then added to both samples and incubated overnight at 37°C. The digestion was stopped by addition of 2 volumes of 5% formic acid: CH_3_CN (1∶1, vol/vol), and dried by vacuum centrifugation. Dried pellets were dissolved in 0.1% TFA in water and the peptides separated from residual salts by binding and elution from C18 ZipTips (EMD Millipore, MA).

Peptides were analyzed by MALDI TOF MS, using a Voyager DE-STR mass spectrometer (ABSciex), with α-cyano 4-hydroxy cinnamic acid as the matrix. Samples were also analyzed by LC/MS/MS, using a 6520 Q-TOF LC/MS mass spectrometer (Agilent, CA). Peptides were injected onto a C18 column (1×50 mm) at 30°C equilibrated with 95% Solvent A (0.1% formic acid in water) and 5% Solvent B (0.1% formic acid in CH3CN). Peptides were eluted with a flow rate of 0.1 mL/min, using a linear gradient to 25% B in 2 min, followed by a linear gradient to 75% B in 15 min. MS spectra were collected in Auto MS2 mode, with a mass range of 300–2500 m/z for MS scans, and 50–3000 m/z for MS2 scans.

MS/MS spectra were analyzed using Mascot v 2.4 (Matrix Science, Inc., MA), searching the human subset of the SwissProt database (http://us.expasy.org/sprot). Search parameters included carbamidomethyl Cys as a fixed modification, and Asn/Gln deamidation as a variable modification. The Mascot DAT output file was then analyzed using Scaffold (Proteome Software). To identify glycopeptides present in these digests, MS/MS fragmentation spectra were examined for common carbohydrate oxonium fragment ions. Mass lists of calculated glycopeptide masses for these fragmentation spectra were entered into GlycoMod (http://web.expasy.org/glycomod/) to identify observed masses of the combined peptide and carbohydrate. Fragmentation spectra were then examined for diagnostic ions to verify both peptide and carbohydrate structure.

### Molecular Modeling

Models of human hTyrC_tr_ and full-length tyrosinases from other species were obtained by homology modeling using the bacterial tyrosinase as a structural template (PDB file: 3nm8). Multiple sequence alignment was performed [50] and integrated in the program Look, version 3.5.2 [51], [52]. The functional domains of human tyrosinase were localized using SMART [53], [54]. The monomeric human tyrosinase structure was built by the automatic segment matching using Look [55], followed by 500 cycles of energy minimization. The conformation of the missense variants, R422Q and R422W, was generated by the same program implicating a self-consistent ensemble optimization (500 cycles) [52]. Finally, the quality of the predicted structure was tested with Procheck [56]. Visualization of 3D structures and computer simulations were with UCSF Chimera [57] and a molecular visualization, modeling, and dynamics program Yasara [58]–[60].

### Statistical Analysis

The experiments were performed in triplicates and error bars represent a standard deviation (SD) from the mean. Michaelis constant, *V*_max_, *k*_cat_ values and other kinetics parameters were averaged and standard errors were determined in each case. The Student’s t-test was applied to evaluate the presence of significant differences between catalytic efficiencies at 37 and 31°C (*p<0.05, **p<0.01). Statistics were generated using the t-test functions available in Excel spreadsheets (Microsoft Office, WA).

## Supporting Information

Figure S1 **Western Blot and SDS-PAGE showing the protein expression of recombinant gene of the intra-melanosomal domain of human tyrosinase in SF9 cells and POV larvae.** **A:** Wild type recombinant hTyrC_tr_. From the left: L, protein ladder; lines 1 and 2, expressed in Sf9 cells hTyrC_tr_ stained with anti-His tag antibody (1∶2000 dilution, EMD Millipore Bioscience Products, MA): 1, 2, BV-1, BV-2, respectively, are two different baculoviruses expressing the same hTyrC_tr_ protein; lines 3 and 4, expressed in POV larvae hTyrC_tr_ stained with anti-His tag antibody (1∶2000 dilution, EMD Millipore Bioscience Products, MA); 3, total homogenate prepared at 1∶10 w/v; 4, supernatant after 13,000×g centrifugation. **B**: Temperature-sensitive mutant variants R422Q and R422W. From the left: L, protein ladder; lines 1 and 2, R422Q stained with anti-tyrosinase T311 antibody (1∶4500 dilution, Santa Cruz Biotechnology, CA) and anti-His antibody (1∶2000 dilution, EMD Millipore Bioscience Products, MA), respectively; lines 3 and 4, R422W stained with anti-tyrosinase T311 antibody (1∶4500 dilution, Santa Cruz Biotechnology, CA) and anti-His antibody (1∶2000 dilution, EMD Millipore Bioscience Products, MA), respectively. **C:** SDS-PAGE of N-glycosyled protein. From the left: L, protein ladder; 1, total lysate; 2, hTyrC_tr_ in presence of PNGase F. Multiple polypeptide bands are derived from the N-glycosylation (Lane 1). The treatment by the PNGase-F shows a strong single band of protein and a weaker band of PNGase-F (Lane 2).
(JPG)

Figure S2 **Temperature and pH dependences of protein activity are shown for hTyrC_tr_ and R422Q, R422W mutant variants.** Optimum temperature for the monophenolase (**A**; L-tyrosine at 0.2 mM) and diphenol oxidase (**C**; L-DOPA at 1.5 mM) activity of hTyrC_tr_ (blue), R422Q (red), and R422W (green) was measured in 50 mM sodium phosphate buffer, pH 7.5 after 30 min of incubation at temperature points: 16, 21, 26, 31, 37, 42, 48, 54, and 60°C. Optimum pH for the monophenolase (**B**; L-tyrosine at 0.2 mM) and diphenol oxidase (**D**; L-DOPA at 1.5 mM) activity of hTyrC_tr_ (blue), R422Q (red), and R422W (green) was measured in 50 mM sodium phosphate buffer after 30 min of incubation at 37°C, pH: 5.0, 5.5, 6.0, 6.5, 7.0, 7.5, 8.0, 8.5, and 9.0. All 490 nm absorbance values are shown after the blank subtraction. Experiments were performed in triplicates and error bars represent standard deviations.
(JPG)

Figure S3 **Inhibition and activation of hTyrC_tr_.** **A–C**: Inhibitory effect of kojic acid, NaCl, and arbutin on monophenolase (0.2 mM L-tyrosine as a substrate) and diphenol oxidase (1.5 mM L-DOPA as a substrate) activity of hTyrC_tr_ is shown by blue and dark magenta colors, respectively. **D**: Effect of HAA on monophenolase and diphenol oxidase activity of hTyrC_tr_ is shown by blue and dark magenta bars, respectively. Both activities were measured in 50 mM sodium phosphate buffer, pH 7.5 after 30 min of incubation with inhibitors/activator at 37°C. Protein concentration 0.5 and 0.05 mg/ml for monophenolase and diphenol oxidase activity, respectively, was used.
(JPG)

Figure S4 **N-linked oligosaccharides from hTyrC_tr_ and two mutants, R422Q and R422W.** Panel **A** shows diphenol oxidase activity of hTyrC_tr_ and two mutants, R422Q and R422W. Glycosylated and deglycosylated proteins are shown by solid and open bars, respectively. **B**: Corresponding Western blots bands obtained with T311 antibody (Santa Cruz Biotechnology, CA). From the left: L, protein ladder; 1, hTyrC_tr_, 2, hTyrC_tr_ in the presence of Endoglycosidase F1; 3, R422Q; 4, R422Q in the presence of Endoglycosidase F1; 5, R422W; 6, R422W in the presence of Endoglycosidase F1. Protein samples were obtained as in Methods section and purified using His-Trap Crude chromatography column (GE HealthCare, NJ). Protein samples were deglycosylated under native conditions by overnight incubation with Endoglycosidase F1 at RT using the Native Protein Deglycosylation Kit (Sigma, MO).
(JPG)

Figure S5 **Protein secondary structure: α-helix and β-sheet content in hTyrC_tr_ and temperature sensitive mutant variants R422Q/W.** Percent of α-helical (**A, B**) and β-sheet (**C, D**) predicted secondary structures for hTyrC_tr_ and mutants R422Q/W shown by blue, red, and green bars, respectively. All calculations were performed in the presence or the absence of 0.5 mM tyrosine at 37°C and 31°C and shown in (**A, C**) and right (**B, D**) panels, respectively. Secondary structure content was calculated using the DICHROWEB web server (http://www.cryst.bbk.ac.uk/cdweb); *p<0.05; ** p<0.001.
(JPG)

Table S1 **Molecular weight of glycosylated hTyrC_tr_ determined by sedimentation equilibrium.**
(JPG)

Table S2 **Detection of N-glycosylation sites by Asn-deamidation after PNGase F treatment. A.** Tyrosinase deglycosylated (with PNGase F). **B.** Tyrosinase control (without PNGase F).
(JPG)

Table S3 **Identification of N-linked glycopeptide compositions.**
(JPG)
