# Supplementary figures and images for: Albinism-Causing Mutations in Recombinant Human Tyrosinase Alter Intrinsic Enzymatic Activity
Source: PLoS One. 2014 Jan 2;9(1):e84494. doi: 10.1371/journal.pone.0084494 (PMC3879332; doi:10.1371/journal.pone.0084494)

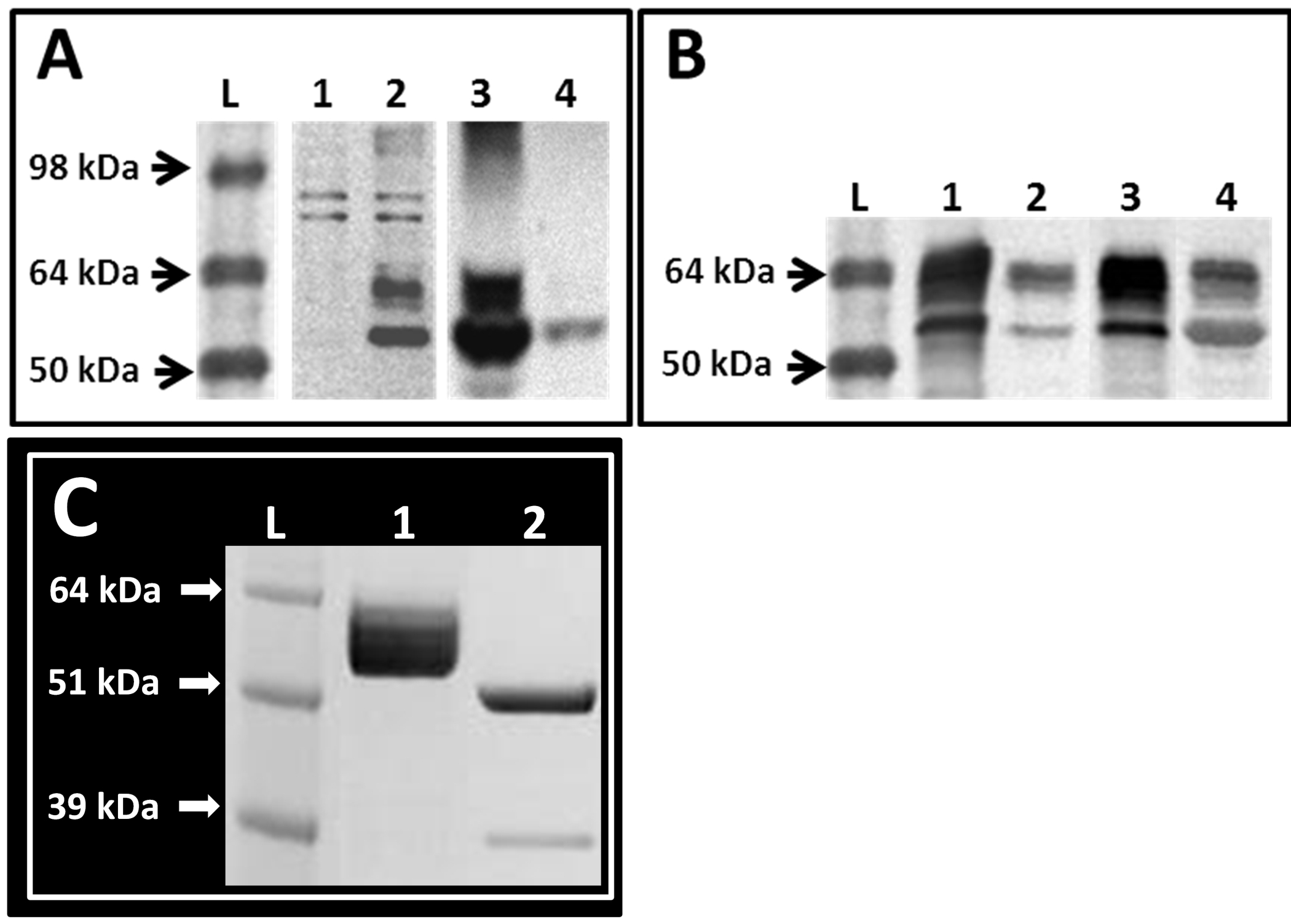

Supplement: Figure S1 — Western Blot and SDS-PAGE showing the protein expression of recombinant gene of the intra-melanosomal domain of human tyrosinase in SF9 cells and POV larvae. A: Wild type recombinant hTyrCtr. From the left: L, protein ladder; lines 1 and 2, expressed in Sf9 cells hTyrCtr stained with anti-His tag antibody (1∶2000 dilution, EMD Millipore Bioscience Products, MA): 1, 2, BV-1, BV-2, respectively, are two different baculoviruses expressing the same hTyrCtr protein; lines 3 and 4, expressed in POV larvae hTyrCtr stained with anti-His tag antibody (1∶2000 dilution, EMD Millipore Bioscience Products, MA); 3, total homogenate prepared at 1∶10 w/v; 4, supernatant after 13,000×g centrifugation. B: Temperature-sensitive mutant variants R422Q and R422W. From the left: L, protein ladder; lines 1 and 2, R422Q stained with anti-tyrosinase T311 antibody (1∶4500 dilution, Santa Cruz Biotechnology, CA) and anti-His antibody (1∶2000 dilution, EMD Millipore Bioscience Products, MA), respectively; lines 3 and 4, R422W stained with anti-tyrosinase T311 antibody (1∶4500 dilution, Santa Cruz Biotechnology, CA) and anti-His antibody (1∶2000 dilution, EMD Millipore Bioscience Products, MA), respectively. C: SDS-PAGE of N-glycosyled protein. From the left: L, protein ladder; 1, total lysate; 2, hTyrCtr in presence of PNGase F. Multiple polypeptide bands are derived from the N-glycosylation (Lane 1). The treatment by the PNGase-F shows a strong single band of protein and a weaker band of PNGase-F (Lane 2). (JPG) [file pone.0084494.s001.jpg]

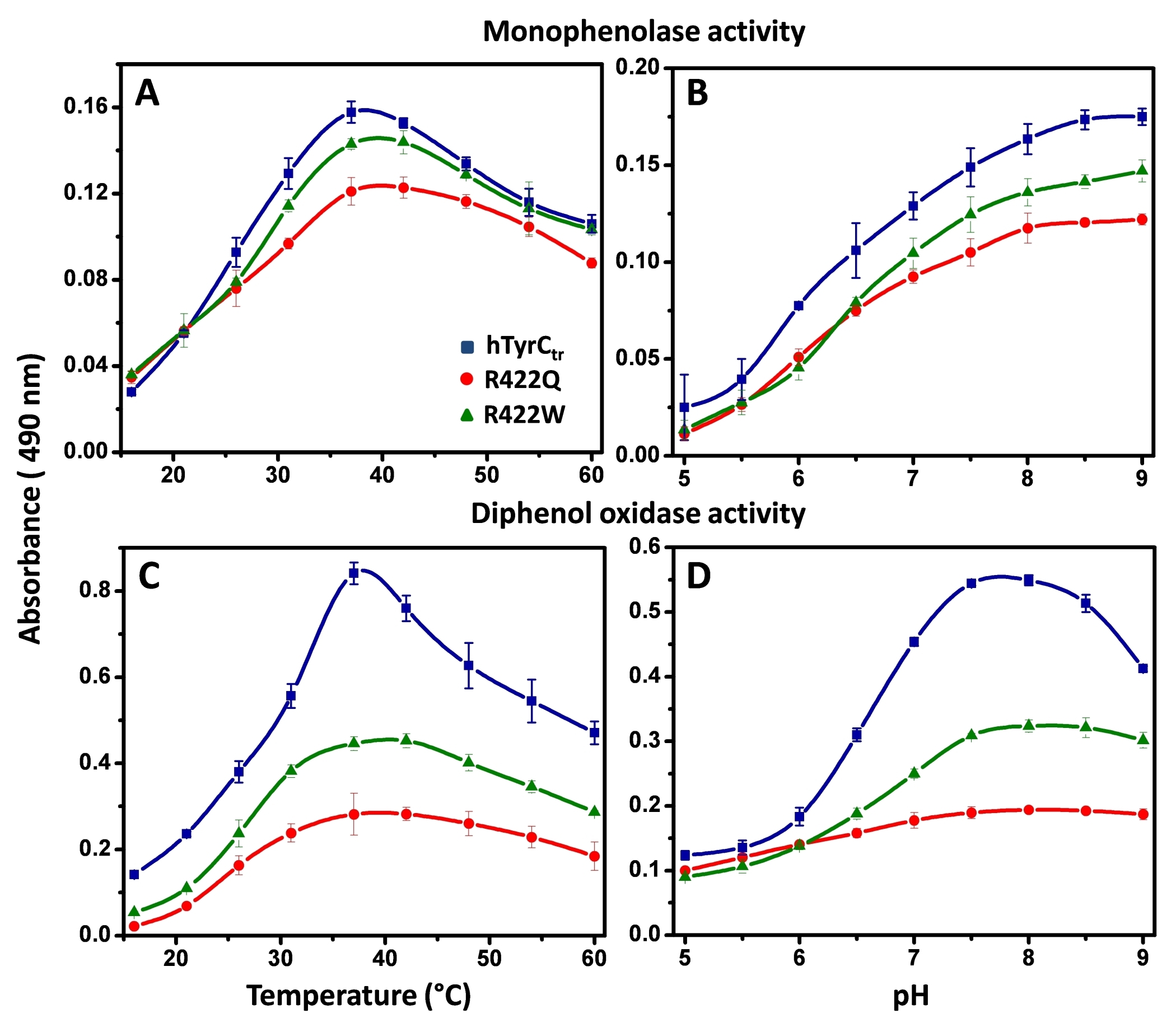

Supplement: Figure S2 — Temperature and pH dependences of protein activity are shown for hTyrCtr and R422Q, R422W mutant variants. Optimum temperature for the monophenolase (A; L-tyrosine at 0.2 mM) and diphenol oxidase (C; L-DOPA at 1.5 mM) activity of hTyrCtr (blue), R422Q (red), and R422W (green) was measured in 50 mM sodium phosphate buffer, pH 7.5 after 30 min of incubation at temperature points: 16, 21, 26, 31, 37, 42, 48, 54, and 60°C. Optimum pH for the monophenolase (B; L-tyrosine at 0.2 mM) and diphenol oxidase (D; L-DOPA at 1.5 mM) activity of hTyrCtr (blue), R422Q (red), and R422W (green) was measured in 50 mM sodium phosphate buffer after 30 min of incubation at 37°C, pH: 5.0, 5.5, 6.0, 6.5, 7.0, 7.5, 8.0, 8.5, and 9.0. All 490 nm absorbance values are shown after the blank subtraction. Experiments were performed in triplicates and error bars represent standard deviations. (JPG) [file pone.0084494.s002.jpg]

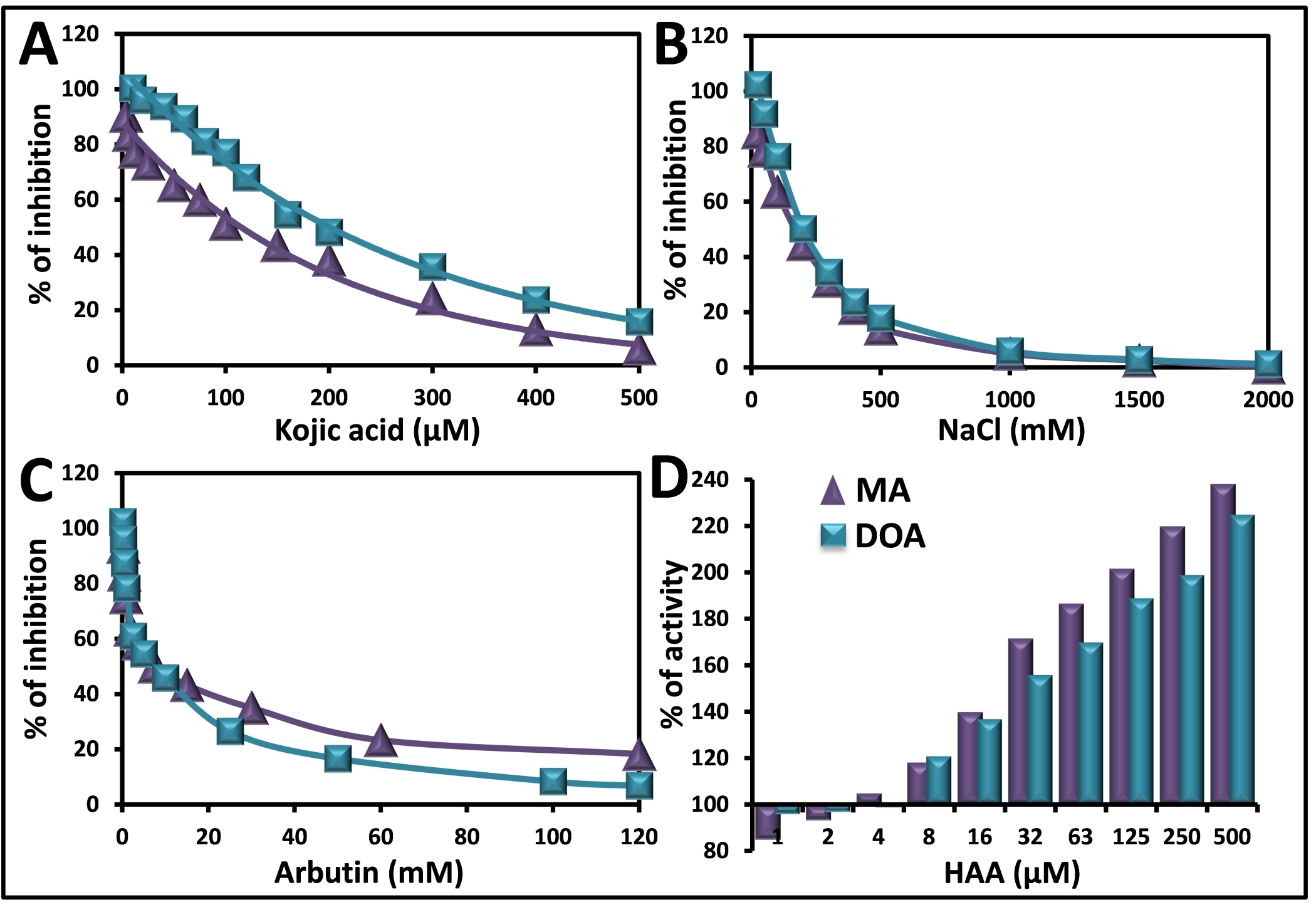

Supplement: Figure S3 — Inhibition and activation of hTyrCtr. A–C: Inhibitory effect of kojic acid, NaCl, and arbutin on monophenolase (0.2 mM L-tyrosine as a substrate) and diphenol oxidase (1.5 mM L-DOPA as a substrate) activity of hTyrCtr is shown by blue and dark magenta colors, respectively. D: Effect of HAA on monophenolase and diphenol oxidase activity of hTyrCtr is shown by blue and dark magenta bars, respectively. Both activities were measured in 50 mM sodium phosphate buffer, pH 7.5 after 30 min of incubation with inhibitors/activator at 37°C. Protein concentration 0.5 and 0.05 mg/ml for monophenolase and diphenol oxidase activity, respectively, was used. (JPG) [file pone.0084494.s003.jpg]

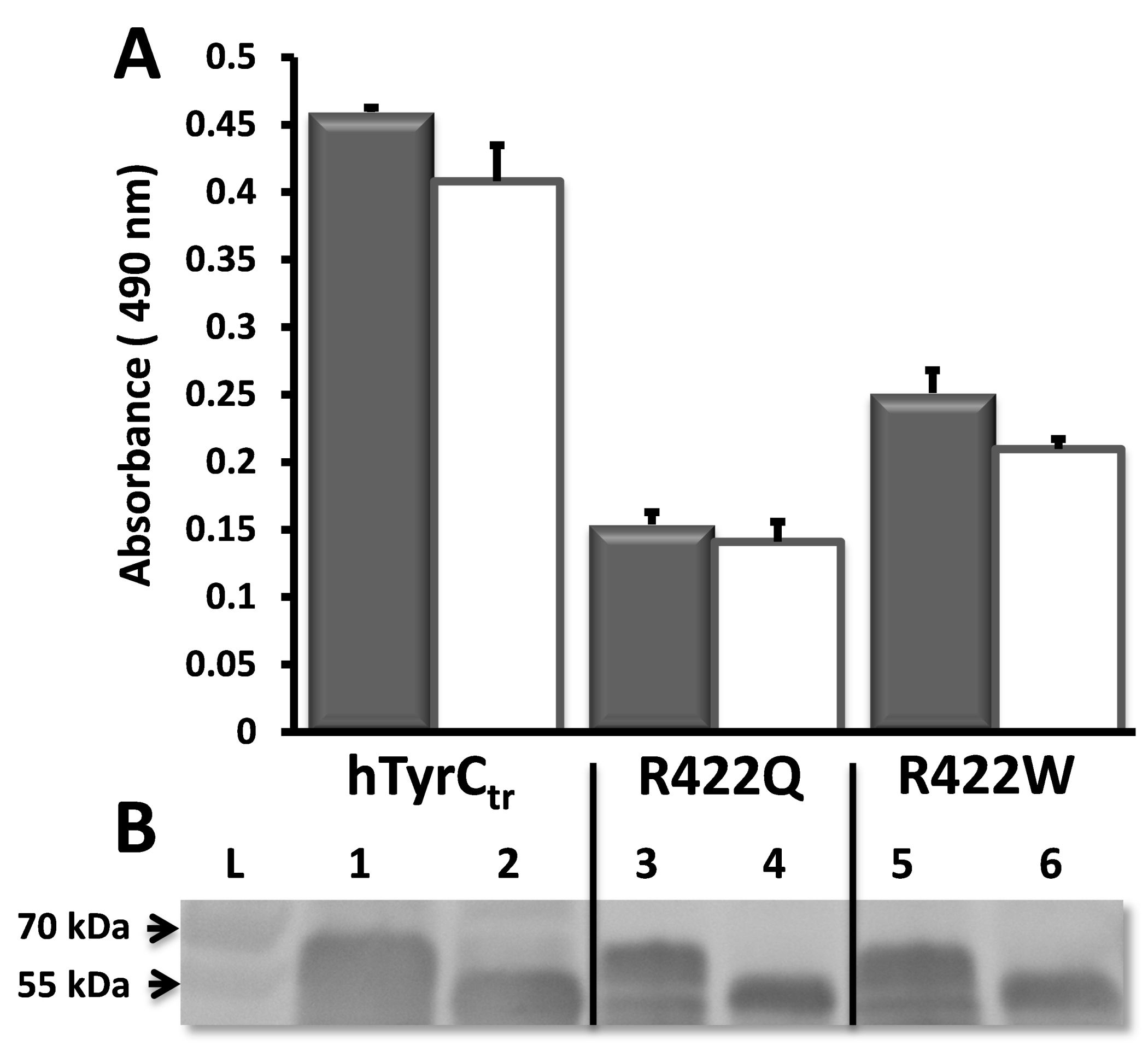

Supplement: Figure S4 — N-linked oligosaccharides from hTyrCtr and two mutants, R422Q and R422W. Panel A shows diphenol oxidase activity of hTyrCtr and two mutants, R422Q and R422W. Glycosylated and deglycosylated proteins are shown by solid and open bars, respectively. B: Corresponding Western blots bands obtained with T311 antibody (Santa Cruz Biotechnology, CA). From the left: L, protein ladder; 1, hTyrCtr, 2, hTyrCtr in the presence of Endoglycosidase F1; 3, R422Q; 4, R422Q in the presence of Endoglycosidase F1; 5, R422W; 6, R422W in the presence of Endoglycosidase F1. Protein samples were obtained as in Methods section and purified using His-Trap Crude chromatography column (GE HealthCare, NJ). Protein samples were deglycosylated under native conditions by overnight incubation with Endoglycosidase F1 at RT using the Native Protein Deglycosylation Kit (Sigma, MO). (JPG) [file pone.0084494.s004.jpg]

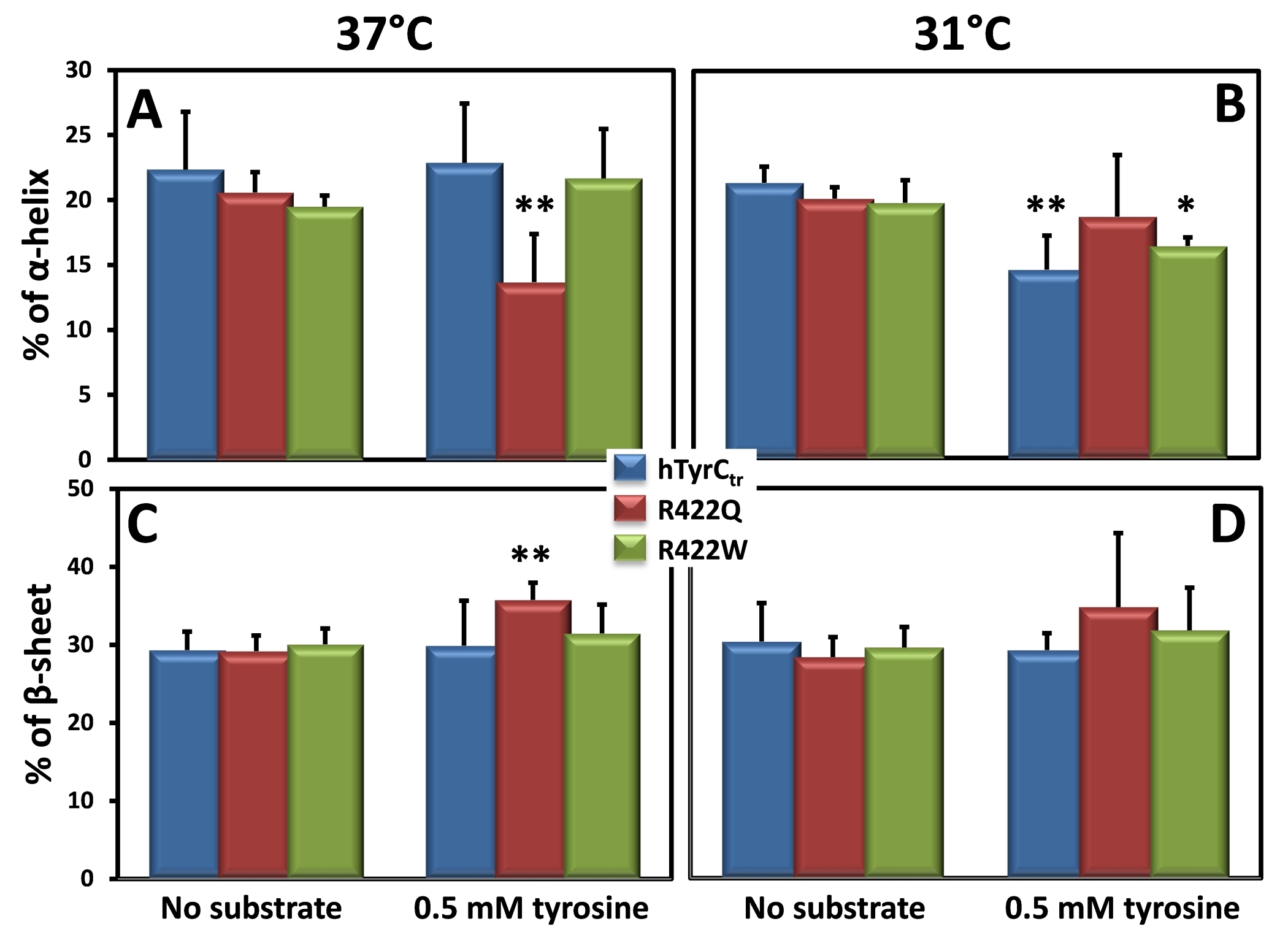

Supplement: Figure S5 — Protein secondary structure: α-helix and β-sheet content in hTyrCtr and temperature sensitive mutant variants R422Q/W. Percent of α-helical (A, B) and β-sheet (C, D) predicted secondary structures for hTyrCtr and mutants R422Q/W shown by blue, red, and green bars, respectively. All calculations were performed in the presence or the absence of 0.5 mM tyrosine at 37°C and 31°C and shown in (A, C) and right (B, D) panels, respectively. Secondary structure content was calculated using the DICHROWEB web server (http://www.cryst.bbk.ac.uk/cdweb); *p<0.05; ** p<0.001. (JPG) [file pone.0084494.s005.jpg]

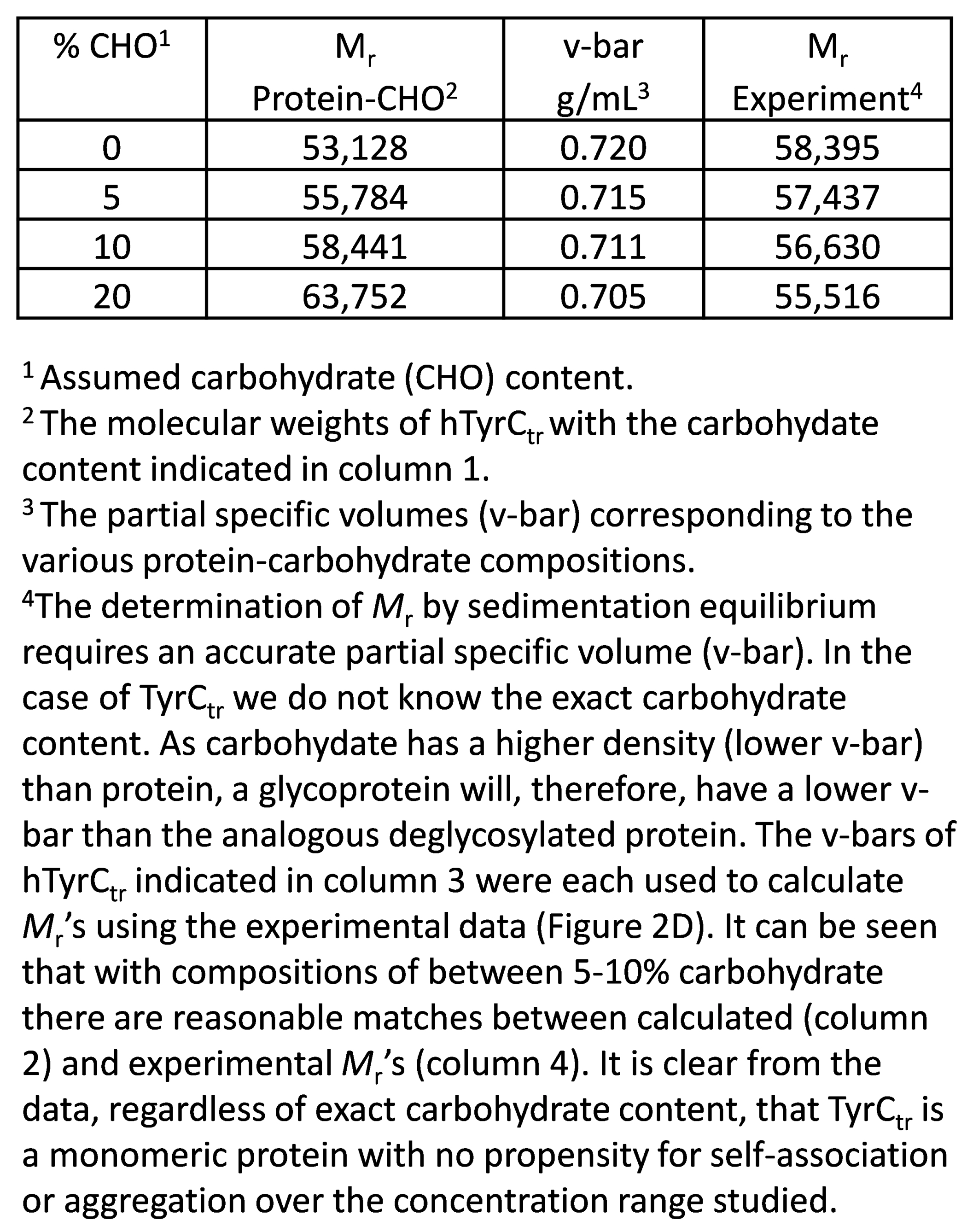

Supplement: Table S1 — Molecular weight of glycosylated hTyrCtr determined by sedimentation equilibrium. (JPG) [file pone.0084494.s006.jpg]

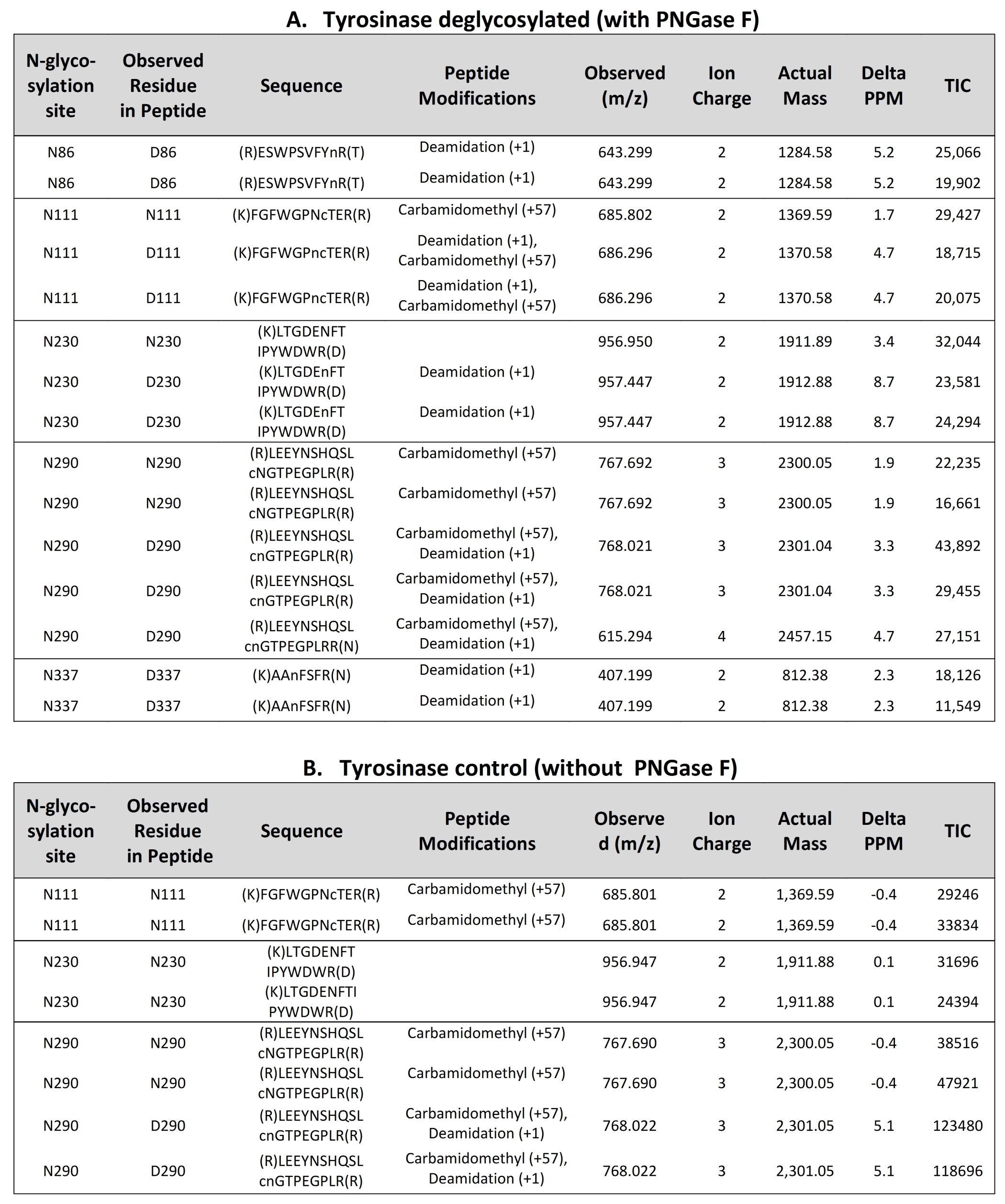

Supplement: Table S2 — Detection of N-glycosylation sites by Asn-deamidation after PNGase F treatment. A. Tyrosinase deglycosylated (with PNGase F). B. Tyrosinase control (without PNGase F). (JPG) [file pone.0084494.s007.jpg]

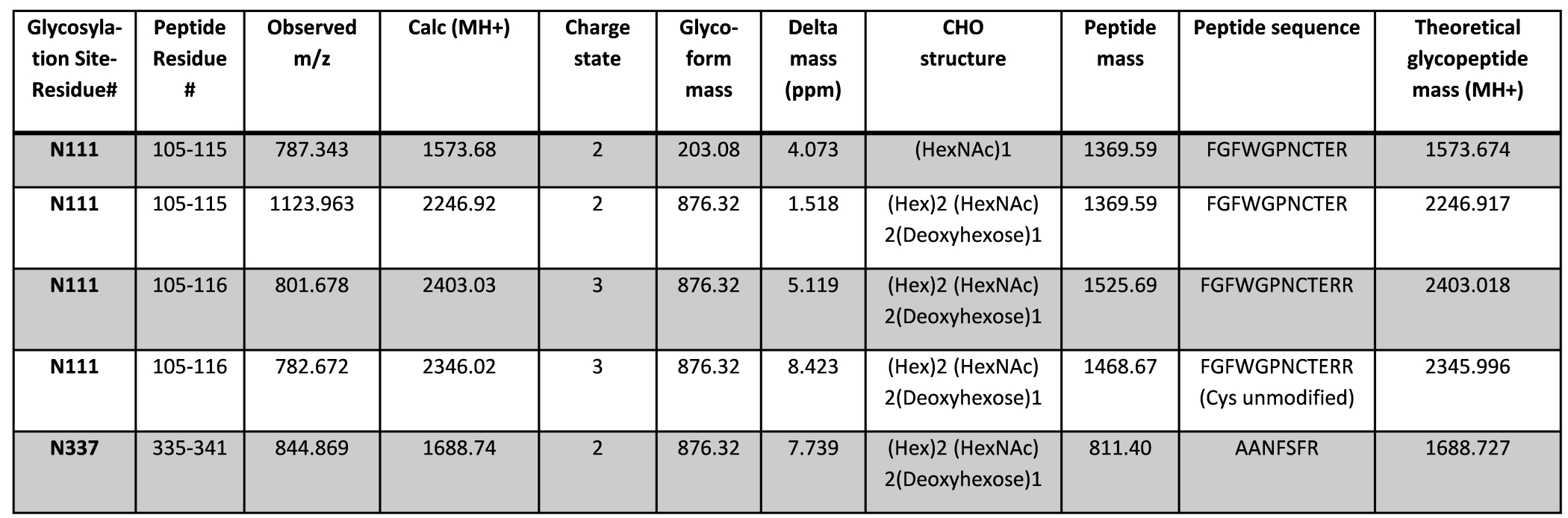

Supplement: Table S3 — Identification of N-linked glycopeptide compositions. (JPG) [file pone.0084494.s008.jpg]
